# Senescence Rewires Microenvironment Sensing to Facilitate Antitumor Immunity

**DOI:** 10.1158/2159-8290.CD-22-0528

**Published:** 2022-10-27

**Authors:** Hsuan-An Chen, Yu-Jui Ho, Riccardo Mezzadra, Jose M. Adrover, Ryan Smolkin, Changyu Zhu, Katharina Woess, Nicholas Bernstein, Georgia Schmitt, Linda Fong, Wei Luan, Alexandra Wuest, Sha Tian, Xiang Li, Caroline Broderick, Ronald C. Hendrickson, Mikala Egeblad, Zhenghao Chen, Direna Alonso-Curbelo, Scott W. Lowe

**Affiliations:** 1 Department of Cancer Biology and Genetics, Memorial Sloan Kettering Cancer Center, New York, New York.; 2 Louis V. Gerstner Jr. Graduate School of Biomedical Sciences, Memorial Sloan Kettering Cancer Center, New York, New York.; 3 Cold Spring Harbor Laboratory, Cold Spring Harbor, New York.; 4 Institute for Research in Biomedicine (IRB Barcelona), The Barcelona Institute of Science and Technology (BIST), Barcelona, Spain.; 5 Calico Life Sciences, South San Francisco, California.; 6 Microchemistry and Proteomics Core Laboratory, Memorial Sloan Kettering Cancer Center, New York, New York.; 7 Howard Hughes Medical Institute, Chevy Chase, Maryland.

## Abstract

Cellular senescence involves a stable cell-cycle arrest coupled to a secretory program that, in some instances, stimulates the immune clearance of senescent cells. Using an immune-competent liver cancer model in which senescence triggers CD8 T cell–mediated tumor rejection, we show that senescence also remodels the cell-surface proteome to alter how tumor cells sense environmental factors, as exemplified by type II interferon (IFNγ). Compared with proliferating cells, senescent cells upregulate the IFNγ receptor, become hypersensitized to microenvironmental IFNγ, and more robustly induce the antigen-presenting machinery—effects also recapitulated in human tumor cells undergoing therapy-induced senescence. Disruption of IFNγ sensing in senescent cells blunts their immune-mediated clearance without disabling the senescence state or its characteristic secretory program. Our results demonstrate that senescent cells have an enhanced ability to both send and receive environmental signals and imply that each process is required for their effective immune surveillance.

**Significance:**

Our work uncovers an interplay between tissue remodeling and tissue-sensing programs that can be engaged by senescence in advanced cancers to render tumor cells more visible to the adaptive immune system. This new facet of senescence establishes reciprocal heterotypic signaling interactions that can be induced therapeutically to enhance antitumor immunity.

See the interview with Direna Alonso-Curbelo, PhD, recipient of the inaugural *Cancer Discovery* Early Career Award: https://vimeo.com/992987447

*See related article by Marin et al., p. 410.*

*This article is highlighted in the In This Issue feature, p. 247*

## Teaser

Tumor cells triggered to senesce alter how they send and receive microenvironmental signals, leading to tissue remodeling, immune cell recruitment, hypersensitization to environmental IFNγ, and improved antitumor immunity.

## INTRODUCTION

Cellular senescence is a stress response program characterized by a stable cell-cycle arrest and a secretory program capable of remodeling the tissue environment (1). In normal tissues, senescence contributes to tissue homeostasis during wound healing; however, in aged or damaged tissues, the aberrant accumulation of senescent cells can cause chronic inflammation and reduced tissue regenerative capacity (2–4). In cancer, senescence has been shown to mediate both beneficial and detrimental effects on tissue biology. On one hand, senescence provides a barrier to oncogene-initiated tumorigenesis and contributes to the antitumor activity of some cancer therapies (5, 6). On the other hand, the persistence of senescent tumor cells after therapy can produce a tissue environment that promotes relapse and metastasis (7, 8). The molecular underpinnings of these opposing biological outputs remain poorly understood.

One facet of the senescence program that is likely to contribute to such diverse biology is the senescence-associated secretory phenotype (SASP; ref. 9). SASP is activated through a global chromatin remodeling process that evolves over time and is controlled by key epigenetic regulators such as BRD4 and proinflammatory transcription factors such as NF-κB and C/EBP-β (10–12). This, in turn, leads to the induction of genes that encode tissue remodeling proteins such as matrix metalloproteinases, growth factors, and fibrolytic factors known to play crucial roles in the would healing process (3, 13, 14). Other SASP components include chemokines and cytokines that can alter the composition and state of immune cells within the tissue, leading to the immune-mediated targeting and clearance of the senescent cells themselves (15, 16). Nonetheless, the aberrant accumulation of senescent cells in many pathologic contexts implies that immune-mediated clearance is not a universal outcome of senescence or the SASP and raises the possibility that additional mechanisms dictate the paradoxically beneficial and detrimental effects of senescence in tissue biology and immune surveillance (17–19).

Certainly, senescence-associated immune surveillance can have potent anticancer effects, though the precise effector mechanisms vary with tissue and cell type (10, 15, 16, 20). In mouse models of hepatocellular carcinoma (HCC), liver tumor cells triggered to senesce are eliminated by immune-dependent mechanisms engaged by wild-type (WT) p53 (15). In agreement, *TP53* is frequently mutated in human HCC, particularly in the “proliferation class” tumors showing the worst prognosis (21, 22). Though immunotherapy and TP53-targeting drugs are emerging as promising strategies to improve disease outcomes, the molecular basis for response and resistance remains unknown (23–25). Therefore, understanding the mechanisms by which senescent liver tumor cells become visible to the immune system may facilitate strategies to elicit antitumor immunity in *TP5*3-mutated HCC that may extend to other tumor types.

Here, we set out to establish principles that modulate the immune recognition and clearance of senescent cells to identify actionable senescence mechanisms that may be exploited to improve the immune control of cancer. To this end, we developed a novel “senescence-inducible” model in which liver cancer cells can be selectively switched to a senescent state through genetic modulation of endogenous p53. We reasoned this would mimic the effects of therapies that trigger senescence (26, 27) while avoiding the confounding effects of senescence-inducing therapies on immune cells or other components of the tissue environment. Using this model and then extending to other systems, we reveal that, in addition to the SASP, senescence drives a major remodeling of the cell-surface proteome and signaling programs in a manner predicted to fundamentally alter the way cells sense and respond to environmental signals, exemplified herein through a hypersensitivity to microenvironmental type II IFN (IFNγ). This process enables a more robust upregulation of the antigen processing and presenting machinery in senescent tumor cells that renders them susceptible to immune surveillance *in vivo*. Thus, our results reveal a rewired tissue-sensing program in senescent cells that acts in concert with SASP to boost their immunogenic potential, thereby facilitating immune-mediated tumor rejection.

## RESULTS

### A p53-Restorable Immunocompetent Tumor Model to Study Senescence Surveillance

**Figure 1.**
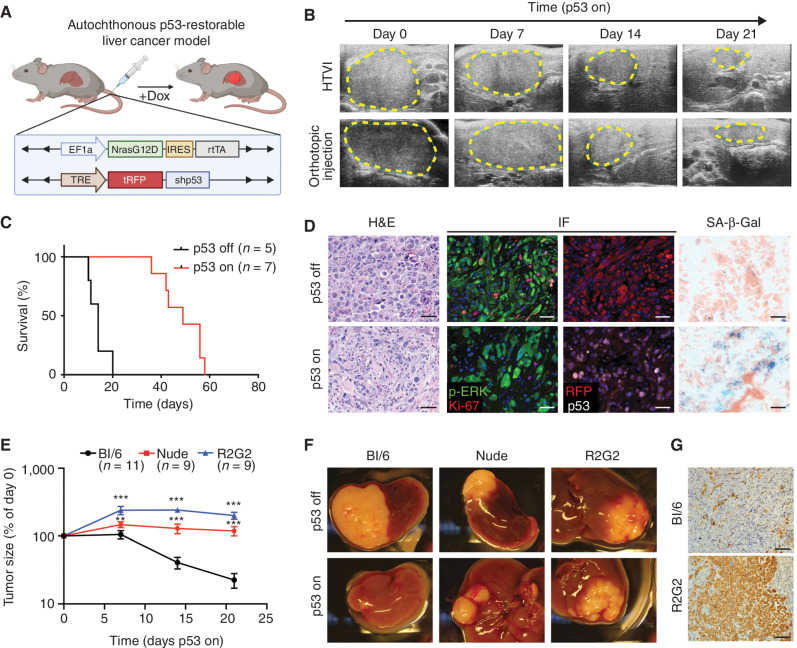
A p53-restorable tumor model to study senescence immune surveillance. **A,** Generation of the p53-restorable, NRAS-driven mouse liver cancer model using the sleeping beauty transposon system delivered through HTVI. (Created with BioRender.com.) **B,** Representative ultrasonogram of HTVI and orthotopic injection liver cancer models at indicated time after p53 restoration. **C,** Survival analysis of mice in the HTVI model. **D,** Representative hematoxylin and eosin (H&E), immunofluorescence (IF), and senescence-associated β-galactosidase (SA-β-gal) staining of p53-suppressed (p53 off) and -restored (p53 on for 14 days) tumor sections generated from the HTVI model. Scale bars, 50 μm. **E–G**, Orthotopic injection of GFP-luciferase vector-transduced NSP tumor cells into the livers of immunocompetent and immunodeficient mouse strains. **E,** Tumor size change measured by ultrasound upon p53 restoration. R2G2, *Rag2-Il2*rg double-knockout mouse. Data are presented as mean ± SEM. *n* e 9 for each strain. **F,** Representative macroscopic pictures at 21 days of p53 on or endpoint p53 off tumor. **G,** Representative IHC staining of GFP-labeled tumor cells at day 21 upon p53 restoration. Scale bars, 100 μm. **, *P* < 0.01; ***, *P* < 0.001.

To study how senescence reprograms cellular and tissue states, we exploited a hydrodynamic tail-vein injection (HTVI) technique (28) to generate a senescence-inducible liver cancer model controlled by a tumor-specific, restorable p53 short hairpin RNA (shRNA). Specifically, adult liver hepatocytes of immunocompetent Bl/6 mice were transfected *in vivo* with a sleeping beauty SB13 transposase vector and two transposon constructs (encoding NrasG12D-IRES-rtTA and TRE-tRFP-shp53, or “NSP”) that integrate in the genome. In this Tet-On system, endogenous p53 is suppressed in the presence of doxycycline (Dox) through the activation of inducible shRNA linked to RFP (Fig. 1A), enabling the genetic control of senescence in established tumors. Consistent with the co-occurrence of mutations that inactivate *TP53* and activate cell proliferation signaling pathways (e.g., PI3K/AKT and RAS/MAPK cascades) in human liver tumors, the cooperation between oncogenic RAS and suppression of p53 led to hepatocyte transformation, with most mice developing tumors with poorly differentiated features 5 to 8 weeks after HTVI. Transcriptional profiling revealed that these murine tumors resemble the “proliferation” class of human HCC (Supplementary Fig. S1A–S1F), which is the typical class of human HCC harboring *TP53* mutations (21, 22, 29).

Based on previous work (15), we anticipated that p53 reactivation in the above system would trigger senescence and engage antitumor immunity. Accordingly, Dox withdrawal triggered dramatic tumor regressions over the course of several weeks, leading to prolonged animal survival (Fig. 1B and C). Analysis of the tumors at 14 days after Dox withdrawal revealed the expected downregulation of the p53 shRNA (as visualized by the linked RFP reporter) and accumulation of senescence-associated β-galactosidase (SA-β-gal) without any notable effects on the RAS-effector p-ERK (Fig. 1D). Similarly, an increase in SA-β-gal activity and SASP-associated transcriptional profiles, together with a concomitant proliferative arrest, was observed in explanted tumor cells 6 to 8 days following p53 restoration (Supplementary Fig. S2A–S2H). Of note, engraftment of these cultures (kept on Dox to maintain p53 silencing) into Dox-fed immunocompetent mice produced synchronous and focal secondary tumors that regressed with similar kinetics as the primary tumors upon Dox withdrawal (Fig. 1B; Supplementary Fig. S3A–S3E). Control experiments using a Tet-Off system or incorporating a constitutive p53 shRNA ruled out the possibility that Dox itself had any effect on tumor behavior in our model (Supplementary Fig. S3F and S3G). Therefore, this system allows for the efficient induction of senescence in tumor cells without resorting to therapies that can also alter the host immune system. Given its added flexibility, we used the orthotopic transplant model (hereafter referred to as “NSP”) for many of the mechanistic studies described below.

As anticipated, the marked tumor regressions noted above were immune mediated. Hence, NSP tumors that arose following transplantation into immunocompromised Nude and *Rag2^‒/‒^Il2rg^‒/‒^* (R2G2) mice underwent a prominent cytostatic response but failed to regress, with R2G2 animals showing the most profound defects (Fig. 1E–G; Supplementary Fig. S3H and S3I). As nude mice are defective in adaptive immunity and R2G2 are also compromised for aspects of innate immunity, these results imply that the adaptive immune system is essential for efficient tumor regression in the model and establish a well-controlled experimental context to explore the mechanistic basis for these effects.

### Senescence Triggers a Switch from Tumor Immune Evasion to Immune Recognition

**Figure 2.**
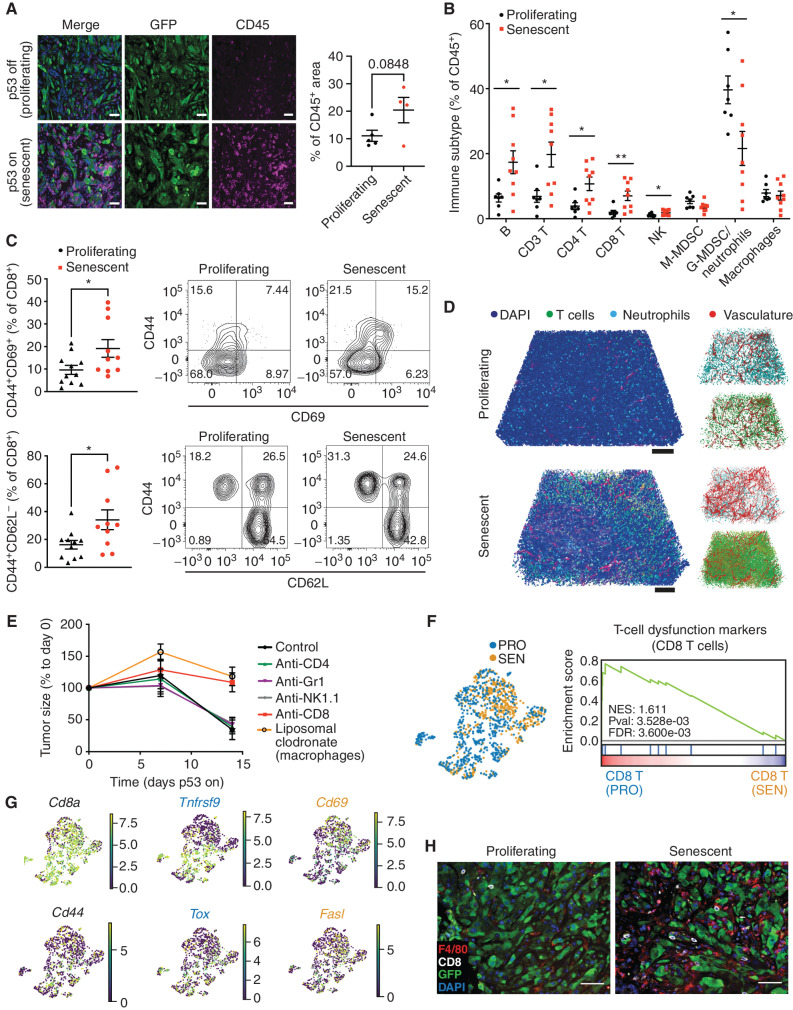
Senescence triggers an immune evasion-to-immune recognition tumor switch. **A,** Representative images of CD45 and GFP staining marking immune cells and tumor cells, respectively, in p53-suppressed and p53-restored tumor (7 days after p53 restoration). Right, the quantification of the area of CD45^+^ staining calculated from 3 random fields per mouse. Each dot represents a mouse. **B,** Flow cytometry analysis of the global immune landscape in an orthotopic NSP liver tumor model. Immunophenotyping of senescent tumors is performed 9 days after Dox withdrawal, a time point when the senescent state is fully established, yet preceding the massive tumor regression. G-MDSC, granulocytic myeloid-derived suppressor cells; M-MDSC, monocytic myeloid-derived suppressor cells. Data are pooled from 2 independent experiments, with *n* = 7 in the proliferating group and *n* = 9 in the senescent group. Note that, as the absolute number of CD45^+^ cells increases in senescent NSP tumor lesions (**A**), so do the total numbers of the indicated cell types. **C,** Flow cytometry analysis of CD8 T cells. Data are pooled from 2 independent experiments, with *n* = 11 in the proliferating and *n* = 10 in the senescent groups. Experiments were performed 9 days after Dox withdrawal. **D,** Representative tissue clearing images of the orthotopic NSP liver tumors. T cells, neutrophils, and vasculature are labeled by CD3, MPO, and CD31 staining, respectively. Samples were collected 9 days after Dox withdrawal. **E,** Tumor size change measured by ultrasound upon p53 restoration in mice after depleting specific immune cell types using antibodies or drugs. **F,** Left, uniform manifold approximation and projection (UMAP) plot of CD8 T cells isolated from p53-suppressed proliferating (PRO) and p53-reactivated senescent (SEN) tumors. Right, gene set enrichment analysis of T-cell exhaustion marker genes in CD8^+^ T cells from proliferating (p53-suppressed) versus senescent (p53-reactivated) tumors. NES, normalized enrichment score; Pval, *P* value. **G,** UMAP plot of the expression of selected genes (*Cd8a*, *Cd44*, *Tnfrsf9*, *Cd69*, *Tox, and Fasl*) between CD8 T cells isolated from senescent (p53-reactivated) and proliferating (p53-suppressed) tumors. **H,** Representative immunofluorescence images of CD8 T cells and F4/80-positive macrophage staining in the orthotopic NSP liver tumor. Tumor samples were collected 9 days after Dox withdrawal. Data are presented as mean ± SEM. All scale bars, 100 μm. A two-tailed Student *t* test was used. *, *P* < 0.05; **, *P* < 0.01.

To characterize the tumor-suppressive paracrine effects of senescence, we next characterized the immune microenvironments of tumors harboring p53-suppressed (referred to as “proliferating”) and p53-restored (referred to as “senescent”) tumor cells after 1 week of Dox withdrawal, a time when senescence is established, but tumors have not yet regressed (Supplementary Figs. S2, S3, and S4A). Lesions harboring senescent tumor cells showed an <1.8-fold increase in total CD45^+^ immune cells compared with proliferating controls (Fig. 2A; refs. 15, 16). Immunophenotypic and histologic analyses (at day 9 after Dox withdrawal) revealed that this involved a prominent increase in the percentage of lymphocytes (B cells, CD4 T cells, and CD8 T cells) and a decrease in the percentage of Gr1^+^ myeloid-derived suppressor cells/neutrophils (CD11b^+^Gr1^+^Ly6C^lo^; Fig. 2B; Supplementary Figs. S4B). Although the fraction of macrophages as a percentage of the total CD45 population remained unchanged, the absolute numbers were markedly increased (Fig. 2A and B; Supplementary Fig. S4C–S4E). Within the T-cell population, accumulating CD8 T cells showed markers of antigen experience (CD44^+^, CD69^+^) and harbored an increased population of effector cells (CD44^+^CD62L^‒^; Fig. 2C; ref. 30). This overall remodeling of the immune environment led to a significant increase in the CD3:neutrophil ratio for tumors harboring senescent cells (Supplementary Fig. S4F), effects consistent with similar increases in the CD3:neutrophil ratio that have been associated with immune reactivity in human liver tumors (31). The remodeling could be clearly visualized using 3D imaging after tissue clearing (Fig. 2D; Supplementary Fig. S4G and S4H; Supplementary Video S1; ref 32).

To pinpoint the specific immune cell types responsible for the immune surveillance of senescent tumor cells, we generated parallel cohorts of mice harboring orthotopic NSP tumors and examined the impact of depleting various immune cell populations on tumor regressions after Dox withdrawal. Whereas blocking antibodies targeting neutrophils/monocytes (Gr1), natural killer (NK) cells (NK1.1), and CD4 T cells (GK1.5) had no effect, depletion of CD8 T cells (2.43) and macrophages (using liposomal clodronate, which selectively targets macrophages (CD11b^+^F4/80^+^) but not classical dendritic cells (CD11b^‒^CD11c^+^MHC^‒^II^+^CD103^+^; refs. 33, 34) markedly impaired tumor regression (Fig. 2E; Supplementary Fig. S4I).

To characterize how p53-driven tumor senescence results in productive antitumor immunity, we performed single-cell RNA-seq (scRNA-seq) analysis of freshly isolated CD45 cells from proliferating and senescent NSP tumors early after Dox withdrawal (8 days; Supplementary Fig. S5A and S5B) and used the differential abundance testing algorithm Milo (35) to capture cell state shifts within the immune cell types mediating this process (Supplementary Fig. S5C–S5F). In line with their contribution to tumor regression, the CD8 T-cell and macrophage subpopulations showed marked changes in quantity and state. Concerning T cells, proliferating (p53-suppressed) tumors were significantly enriched in CD8 T states exhibiting high expression of both dysfunction markers (*Tox, Tigit, Lag3, Ctla4*, *Pdcd1*/*PD1*, and *Cd160*) and activation markers (*Prf1*; Fig. 2F and G; Supplementary Fig. S5G; Supplementary Table S1). These CD8 T cells also showed high levels of *Tnfrsf9*, a marker known to delineate T-cell subsets that have the capacity to become reinvigorated in human HCC and other cancer types (36, 37). In stark contrast, in senescent (p53-reactivated) lesions, CD8 T populations appeared highly activated, showing low levels of dysfunction markers and high expression of effector cytokines (e.g., *Ifng, Tnf*; Supplementary Table S1). Accordingly, the transcriptional profiling of bulk tumor tissues showed immune active and cytotoxic signatures in senescent tumors undergoing regression (Supplementary Fig. S5H; ref. 38).

Changes to the macrophage compartment provided further evidence that tumor cell senescence triggered an immune evasion-to-surveillance switch. Hence, scRNA-seq, immunophenotyping, and histology indicated that tumor-associated macrophage phenotypes transitioned from F4/80^lo^;CD11c^hi^ states (cluster 8), including immune-suppressive PD-L1^+^ populations (characteristic of human HCC tumors with poor prognosis; refs. 39, 40) to F4/80^hi^;CD11c^‒^ states (cluster 0), defined by high expression of an antigen-presentation gene signature (Supplementary Figs. S5E–S5J and S6A and S6B; Supplementary Table S1). Of note, these senescence-associated F4/80^hi^;CD11c^‒^ macrophages were particularly sensitive to the liposomal clodronate treatment (Supplementary Fig. S6C–S6E), which also resulted in a significant reduction in the fraction of active CD8 but not CD4 T cells (Supplementary Fig. S6F and S6G), indicating a CD8 T–dependent immune response involving cooperativity with macrophages. Accordingly, histologic analyses confirmed that accumulating CD8 T cells and F4/80^+^ macrophages were frequently coenriched following senescence induction in tumors (Fig. 2H; Supplementary Fig. S4D). Collectively, these biological and molecular analyses support a model in which tumor cell senescence induces an abrupt switch from immune evasion to immune surveillance mediated by changes in macrophages and CD8 T-cell states, leading to productive antitumor immunity and, ultimately, tumor rejection.

### Senescence Remodels Tissue-Sensing Programs and Cell-Surfaceome Landscape

**Figure 3.**
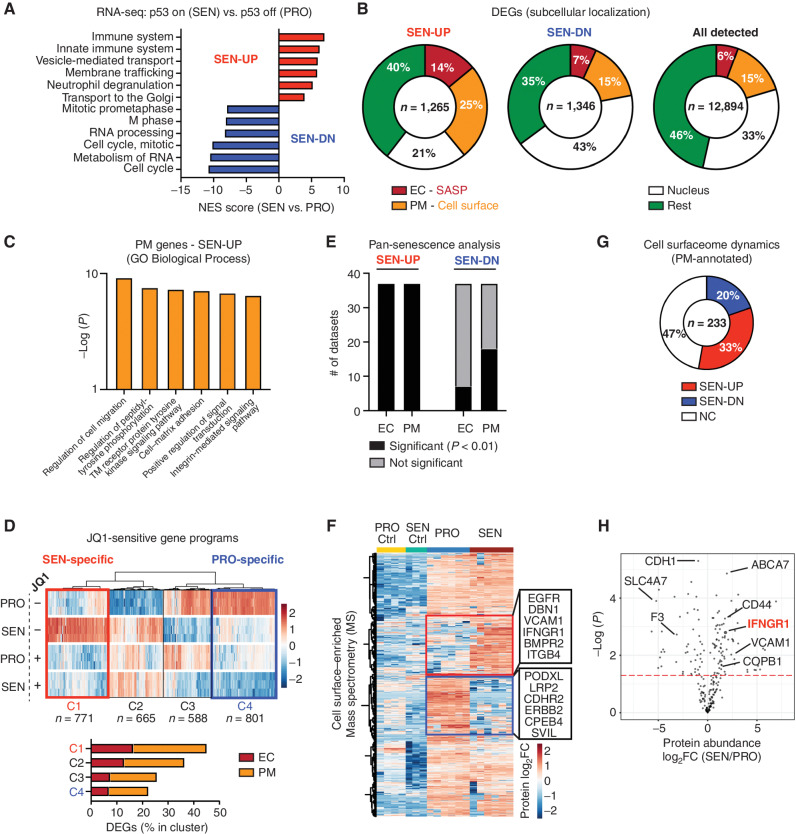
Senescence remodels tissue-sensing programs and cell-surfaceome landscape. **A,** Gene set enrichment analysis (Reactome) of RNA-seq data from proliferating (PRO, p53 off) versus senescent (SEN, p53 on for 8 days) NSP liver tumor cells *in vitro*. NES, normalized enrichment score. **B,** Subcellular localization of DEGs (*P* < 0.05; fold change > 2) in all detected genes [transcripts per kilobase million (TPM) > 1] from RNA-seq. **C,** Gene ontology (GO) analysis of DEGs encoding PM proteins upregulated in senescent cells. TM, transmembrane. **D,** Transcriptomic analysis of all DEGs (proliferating vs. senescent) in the presence or absence of JQ1 treatment. The C1 cluster (in red) contains the senescence-specific genes sensitive to JQ1, and the C4 cluster (in blue) contains the proliferation-specific genes sensitive to JQ1. **E,** Meta-analysis of RNA-seq dataset from SENESCopedia by performing subcellular localization of DEGs (same as Fig. 2D) and Fisher exact test to examine the relative enrichment of upregulated and downregulated EC/PM-DEGs deviated from the random distribution. See also Supplementary Fig. S7E and S7F. **F,** Mass spectrometry (MS) analysis of PM-enriched proteome in proliferating and senescent cells. Protein level is normalized to mean expression of the protein of all samples. Controls are the samples without biotin labeling serving as background. Red and blue boxes represent proteins enriched in senescent and proliferating cells, respectively. *n* = 6 for both the senescent and proliferating experimental groups, and *n* = 3 and 4, respectively, for their control. **G,** Distribution of upregulated and downregulated GeneCards-annotated PM proteins profiled by MS. NC, no change. **H,** Volcano plot of GeneCards-annotated PM proteins profiled by MS.

We next set out to exploit the above model to understand the molecular mechanisms responsible for rendering senescent tumor cells visible to the immune system. Senescence induction involves a chromatin remodeling program that silences proliferative genes and activates many genes encoding SASP factors, with the latter program being largely dependent on the enhancer reader BRD4 (10). We therefore performed transcriptional profiling experiments on NSP cells under proliferating (p53-suppressed) versus senescent (p53-restored) conditions in the absence and presence of JQ1, a drug that inhibits BRD4 function (Supplementary Table S2). Consistent with expectations, p53 restoration dramatically reduced the expression of proliferative genes and induced the expression of well-known SASP factors (Fig. 3A; Supplementary Fig. S7A; ref. 7), including several cytokines known to stimulate T cells (*Cxcl16, Il18*) or macrophage activation and recruitment (*Csf2*, encoding protein GM-CSF) or previously linked to senescence (*Igfbp7, Igfbp3, Pdgfa*). As anticipated from previous work (10), many of the upregulated SASP-encoding transcripts (<65%) were BRD4-dependent (Supplementary Fig. S7B). Similarly, a range of growth factors and immune modulators were secreted from the senescent cells, as assessed by multiplexed cytokine assays, including the T-cell and macrophage attractants CCL5, CXCL9, and GM-CSF, as well as the vasculature remodeling factor VEGF (Supplementary Fig. S7C). Therefore, senescence in p53-restored NSP tumor cells is associated with a robust SASP, consistent with the marked remodeling of the immune ecosystem characterized above.

Strikingly, examination of the subcellular localization for differentially expressed genes (DEG) revealed that senescent tumor cells not only increased their expression of secreted (“extracellular,” EC) SASP factors, but also displayed major changes in the expression levels of transcripts encoding surface proteins (“plasma membrane,” PM; Fig. 3B). Indeed, 25% of total upregulated DEGs encoded PM proteins, a significant enrichment that deviated from the random distribution (15%; Fig. 3B). Dynamic PM-DEGs were linked to protein tyrosine kinase signaling transduction (*Nrp1, Egfr*), cytokine receptor activity (*Ifngr1*), extracellular matrix receptors (*Itgb3*, *Cd44*), and ion transporters (*Slc12a1*, *Slc24a3*) and captured known senescence-associated molecules (*Cd44, Vcam1*, and *Itgb3*), suggesting senescent cells may have an enhanced capability to interact with and sense their environment (Fig. 3C; Supplementary Fig. S7D; refs. 41–43).

Interestingly, the senescence-associated increase in the expression of many of these PM proteins was blunted by JQ1, suggesting that their induction may be part of the broader chromatin remodeling program coupled to SASP (Fig. 3D; ref. 10). Of note, profound changes in the transcription of genes encoding PM proteins also occurred in p53-deficient NSP tumor cells treated with the senescence-inducing drug combination trametinib and palbociclib (Supplementary Fig. S7E, top panel; Supplementary Table S2; ref. 20) and in a series of 13 genetically diverse *TP53* WT and *TP53*-mutant human cancer lines derived from liver, breast, lung, and colon cancers induced to senesce by various triggers (Fig. 3E; Supplementary Fig. S7F; ref. 44). This was particularly robust for upregulated (but not downregulated) PM-DEGs, eminiscent of effects observed for EC SASP factors (Fig. 3E; Supplementary Fig. S7E, bottom panel). Therefore, the markedly altered expression of cell-surface proteins we observed in our model extends beyond p53-induced senescence and may be a hallmark of the senescent state.

To validate the global remodeling of PM factors in senescence at the protein level, we performed surface proteomics on isogenic proliferating and senescent NSP tumor cells, using a biotin-labeling enrichment method, in which cell-surface proteins were labeled with membrane-impermeable biotin, purified, and subjected to mass spectrometry (Fig. 3F; Supplementary Fig. S7G; ref. 45). A strong correlation between biological replicates under each condition was observed (Supplementary Fig. S7H), with detected proteins being enriched for annotated PM proteins by 60% after induction of p53-induced senescence. Of 887 proteins that were reproducibly detected, more than 50% were differentially expressed. Most differentially expressed proteins correlated well with the directionality observed in our transcriptional profiling data, although some were differentially expressed without a corresponding change in transcript levels (Supplementary Fig. S7I).

Annotated cell-surface proteins detected by mass spectrometry upon senescence induction included several previously linked to senescence (e.g., CD44 and VCAM1), various growth factor and cytokine receptors (e.g., EGFR, ICAM1, and IFNGR1), and other less characterized factors (Fig. 3F–H; Supplementary Fig. S7J and S7K). Of note, the set of cell surface–enriched proteins identified in our model showed limited overlap with those identified in human fibroblasts undergoing oncogene-induced senescence (46), suggesting heterogeneity between cell types or senescence triggers. Regardless, these results show that in addition to a rewiring in their secretory program, senescent cells undergo profound changes in the content and abundance of cell-surface proteins and imply that senescent cells acquire distinctive microenvironment-sensing traits that may influence their state and fate *in vivo*.

### Senescent Cells Are Primed to Sense IFNγ and Amplify IFNγ Signaling

**Figure 4.**
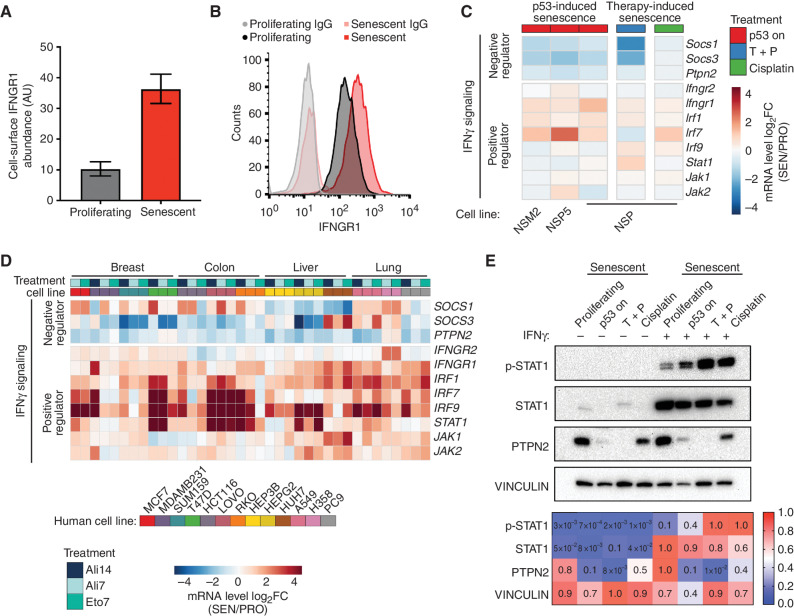
Senescent cells are primed to sense and amplify IFNγ signaling. **A** and **B,** IFNGR1 level on proliferating and senescent cells profiled by mass spectrometry (**A**) and validated by flow cytometry (**B**). AU, arbitrary unit. Data are presented as mean ± SEM. *n* = 6 for both the proliferating and senescent groups. **C,** Transcriptomic analysis of selected genes regulating IFNγ signaling from RNA-seq data of 3 independent p53-restorable cell lines (NSP, NSM2, and NSP5) restoring p53 along with NSP cells treated with two other senescence triggers. SEN/PRO, senescent/proliferating; T + P, trametinib plus palbociclib. **D,** mRNA expression of selected genes involved in IFNγ signaling in human cell lines triggered to senesce. Treatment: Ali, alisertib; Eto, etoposide; number indicates the length of treatment (days). Data are obtained from the public dataset SENESCopedia (44). **E,** Top, immunoblot analysis of NSP cells under different senescent triggers in the presence or absence of IFNγ (1 ng/mL). Bottom, quantification of the intensity of signal from immunoblot. p-STAT1, phospho-STAT1 (Tyr701).

To identify pathways that might functionally influence how senescent cells sense their environment, we mined transcriptional and proteomic datasets for senescence-associated changes linked to antitumor immunity. Interestingly, gene ontology (GO) analysis revealed that type II interferon-gamma (IFNγ) response (47) was among the top 5 annotated pathways enriched during senescence and dependent on cell state–specific enhancer programs (i.e., JQ1-sensitive; i.e., “C1” of Fig. 3D; Supplementary Fig. S8A). Among the altered transcripts, we noted several positive regulators of IFNγ signaling, including the IFNγ receptor subunit IFNGR1 (one of the most significantly upregulated proteins from our proteomic data) and multiple interferon effectors (*Irf1*, *Irf7*, and *Irf9*; refs. 47, 48; Fig. 4A–C; Supplementary Fig. S8B and S8C). Besides these Brd4-sensitive–upregulated genes, transcripts encoding negative regulators of IFNγ signaling (*Ptpn2*, *Socs1*, and *Socs3*) were significantly decreased (Fig. 4C; refs. 49, 50). Similar changes were noted in NSP tumor cells treated with different senescence inducers (Fig. 4C; Supplementary Fig. S8D–S8G) and, more broadly, in a panel of 13 human breast-, lung-, liver-, and colon-derived cancer cell lines triggered to senesce (Fig. 4D; ref. 44). Therefore, changes in the expression of type II IFN signaling components are a general feature of senescent cells, independent of cell type, cell genotype, species, and nature of the senescence inducer.

The concurrent increase in IFNγ signaling effectors and decrease in negative regulators led us to hypothesize that senescent cells become primed to sense IFNγ within their environment. To test this hypothesis directly, we treated proliferating and senescent NSP cells with recombinant IFNγ and performed immunoblotting analyses of JAK–STAT signaling activation. Although IFNγ dramatically increased the baseline levels of STAT1 in both states, senescent cells accumulated more phosphorylated STAT1, irrespective of the senescence trigger (Fig. 4E; Supplementary Fig. S8H). Additionally, we also found an increased level of phosphorylated JAK1 in p53-restored senescent cells, further supporting our finding on a more active JAK–STAT signaling pathway in senescent cells sensing IFNγ (Supplementary Fig. S8I). As predicted from transcriptional analyses, senescence also triggered a decrease in PTPN2 protein (51), irrespective of the presence of exogenous IFNγ (Fig. 4E). Thus, senescent cells more efficiently activate IFNγ signaling in response to limiting concentration of IFNγ in the environment.

### Senescence and EC IFNγ Cooperatively Upregulate the Antigen Processing and Presentation Machinery

**Figure 5.**
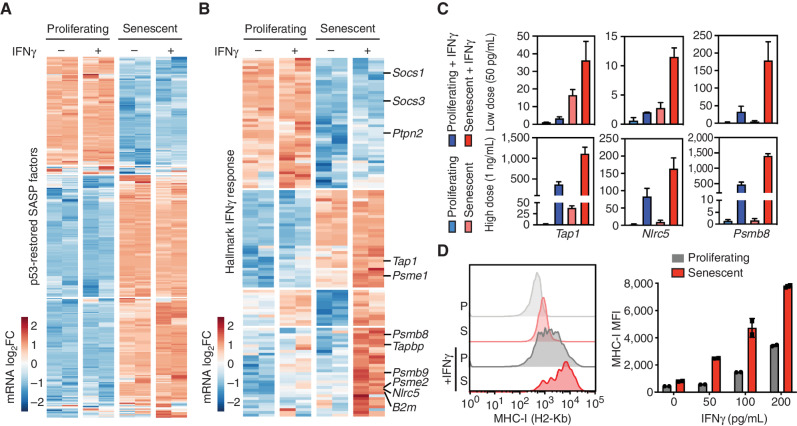
Senescence and EC IFNγ cooperate to upregulate antigen processing and presentation machinery. **A** and **B,** mRNA expression of genes in proliferating and senescent NSP cells *in vitro* in the presence or absence of IFNγ (50 pg/mL) treatment. mRNA level is normalized to the mean expression of the gene in all samples. **A,** DEG-encoding SASP factors in our model. **B,** IFNγ response genes from the Hallmark signature database. **C,** RT-qPCR of selected antigen presentation pathway genes in proliferating and senescent cells treated with low (50 pg/mL) or high (1 ng/mL) concentration of IFNγ. Samples are from 2 biological replicates. **D,** MHC-I level of proliferating and senescent cells treated with IFNγ for 24 hours measured by flow cytometry. MFI, median fluorescence intensity. Data are presented as mean ± SEM.

To better understand the functional contribution of IFNγ sensing to the senescence program, we next compared the phenotypic and transcriptional states of proliferating and p53-restored senescent NSP tumor cells treated with recombinant IFNγ at a low (50 pg/mL) or higher (1 ng/mL) dose. Although the addition of exogenous IFNγ to proliferating or senescent tumor cells had a negligible effect on the viability, proliferation, or SASP gene expression of either cell type at the doses tested (Fig. 5A; Supplementary Fig. S9A–S9D), marked changes in IFNγ pathway gene expression linked to the senescent state were observed. Specifically, supervised clustering of the Hallmark “IFNγ response signature” across proliferating and senescent cells revealed three DEG modules: (i) genes that are downregulated during senescence irrespective of IFNγ (including the aforementioned negative regulators); (ii) genes that are upregulated during senescence irrespective of IFNγ; and, interestingly, (iii) a substantial set of DEGs that are cooperatively induced by the combination of senescence and IFNγ (Fig. 5B). Therefore, senescence triggers quantitative and qualitative changes in the transcriptional response to IFNγ.

One well-established output of IFNγ signaling regulating cells’ susceptibility to adaptive immune surveillance is an increased capacity for antigen presentation mediated by MHC class I molecules (MHC-I; refs. 47, 52). Indeed, many of the genes upregulated in senescent cells (class ii genes) or hyperinduced in the presence of exogenous IFNγ (class iii genes) included components of the antigen presentation machinery. Among the genes induced during senescence (class ii genes) were *Tap1*, transporters associated with antigen processing, and *Psme1*, a proteosome factor associated with antigen processing (53). Those hypersensitive to exogenous IFNγ (class iii genes) included *Nlrc5*, a transcriptional coactivator of MHC-I genes (54); the MHC-I assembly factor *Tapbp*; and the MHC-I subunit *B2m*. Two other class iii genes were components of the immunoproteasome (*Psmb8* and *Psmb9*) whose actions can alter the repertoire of presented peptides when overexpressed and are associated with an improved tumor response to immune-checkpoint blockade (55). This amplified output of IFNγ in senescent cells was confirmed by RT-qPCR and was retained at even higher levels of exogenous IFNγ (Fig. 5C; Supplementary Table S3). Consistent with the multifactorial process described above, this effect was not observed in proliferating tumor cells, even those overexpressing an IFNGR1 cDNA and/or treated with IFNγ (Supplementary Fig. S10A–S10D).

Also consistent with the gene expression changes described above, senescent tumor cells more robustly upregulated MHC-I in response to low levels of exogenous IFNγ compared with proliferating counterparts. Hence, whereas cell-surface levels of MHC-I of both proliferating and senescent cells were low at baseline and induced by exogenous IFNγ, senescent cells showed a significant increase of MHC-I protein expression (Fig. 5D). Similar synergies were observed for cell-surface HLA expression (identical to MHC-I in mice) in human cancer cells from liver and other cancer types triggered to senescence with nutlin, which engages a p53-dependent senescence program (56), or trametinib/palbociclib, which preferentially targets tumor cells with an activated MAPK pathway (Supplementary Fig. S11A–S11D; ref. 20). Of note, the combinatorial effects of drug treatment and IFNγ on HLA expression required senescence induction and did not occur in liver tumor cells that failed to senesce owing to a spontaneous or engineered p53 mutation (irresponsive to nutlin) or a nonhyperactivated MAPK pathway (irresponsive to trametinib/palbociclib). Furthermore, even though type I and II IFN response pathways include overlapping components, exogenous IFNβ treatment could not substitute for IFNγ in producing a robust MHC-I induction in senescent cells nor a strongly differential induction between proliferating and senescent cells in our p53 restoration model (Supplementary Fig. S11E and S11F). These data imply that murine and human cells triggered to senesce acquire an increased capacity for antigen processing and presentation in the presence of limiting quantities of IFNγ.

### Senescent Tumor Cells Hyperactivate the IFNg Signaling Pathway *In Vivo*

**Figure 6.**
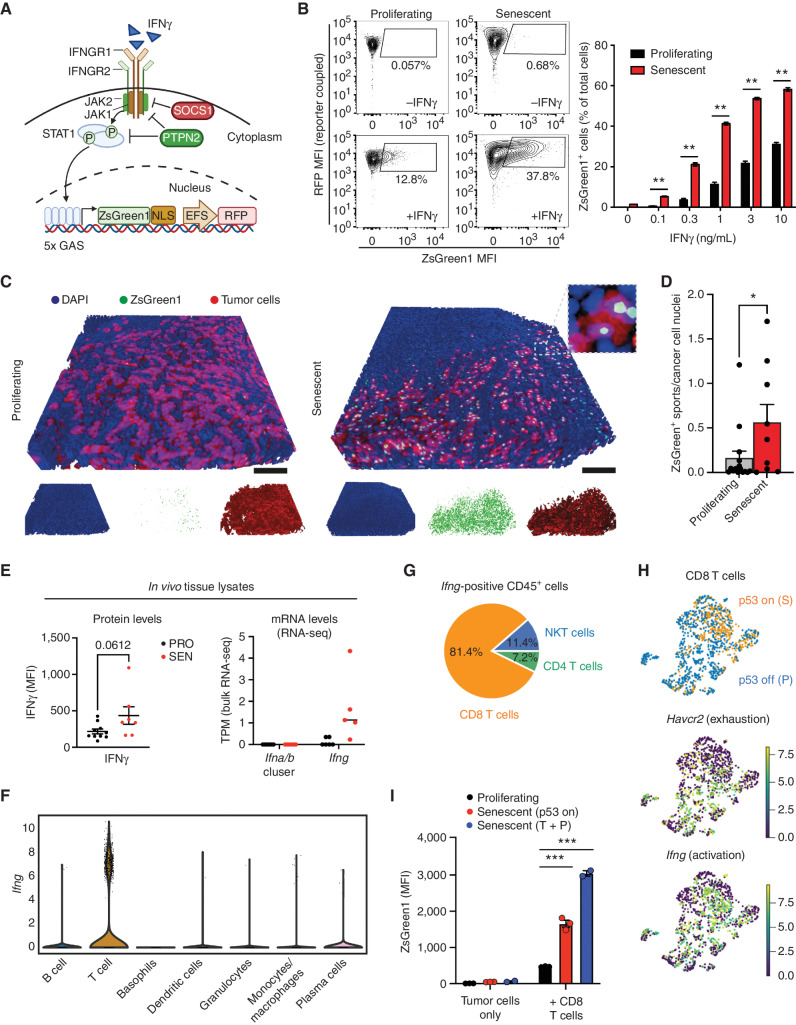
Senescence enhances IFNγ-mediated heterotypic signaling from activated immune cells to tumor cells. **A,** Graphic illustration of the IGS reporter. (Created with BioRender.com.) **B,** Left, representative flow cytometry plots measuring ZsGreen1 signals in proliferating and senescent NSP cells treated with 1 ng/mL IFNγ. Right, quantification of the percentage of ZsGreen1-positive cells upon IFNγ treatment. MFI, median fluorescence intensity. **C** and **D,** Representative 3D imaging of tissue-cleared tumors from the orthotopically injected liver NSP cell line expressing IGS reporter (**C**). Quantification of 3 randomly selected fields from the liver tumor of each mouse (**D**). *n* = 5 and *n* = 3 for the proliferating and senescent groups (9 days after p53 restoration), respectively. Scale bars, 100 μm. **E,** Top, cytometric bead array assay for the IFNγ level from *in vivo* tumor tissue lysate samples (7 days after p53 restoration). Bottom, transcripts of indicated genes from RNA-seq of *in vivo* bulk samples of tumors generated by HTVI (PRO, p53 off; SEN, p53 restoration for 12 days). TPM, transcripts per kilobase million. Noted *Ifna/b* cluster contains 14 *Ifna* subtypes and 1 *Ifnb* gene. **F** and **G,** Expression of Ifng in tumor-infiltrating immune cells profiled by scRNA-seq in the NSP transplantable model (as in Fig. 2, sample collected at day 8 after p53 restoration). **H,** Uniform manifold approximation and projection plot of the expression of *Havcr2* (encoding TIM3) and Ifng in CD8 T cells harvested from proliferating (P) and senescent (S) tumor lesion. Top panel is replicated from Fig. 2F (left) to indicate cells corresponding to each condition. **I,** Quantification of ZsGreen1 intensity of NSP tumor cells in the OT-I T-cell and SIINFEKL-expressing tumor cell coculture experiment (effector-to-target ratio, 5:1) after 20 hours of coculture. Signal measured by flow cytometry. T + P, trametinib plus palbociclib. See experimental details in Supplementary Fig. S12E. Data are presented as mean ± SEM. Two-tailed Student *t* test was used. *, *P* < 0.05; **, *P* < 0.01; ***, *P* < 0.001.

To determine the *in vivo* consequences of the rewiring of IFNγ signaling identified in senescent cells, we next adapted an IFNγ sensing (IGS) reporter system to directly visualize intracellular IFNγ signaling activation in real time (57). This reporter consists of a series of consensus IFNγ-activated sequences, which has specificity to type II IFN over other signals (57), followed by a cDNA sequence encoding ZsGreen1 fluorescent protein and is linked to a constitutively expressed RFP transgene to visualize transduced cells (Fig. 6A). NSP tumor cells expressing this construct were RFP positive and showed a dose-dependent increase in ZsGreen1 signal upon treatment with IFNγ *in vitro* that increased following p53 induction or following treatment with senescence-inducing drugs (Fig. 6B; Supplementary Fig. S12A and S12B).

We next used this system to monitor signaling activity following senescence induction in tumors. Reporter-transduced tumor cells (on Dox) expressing constitutive RFP were injected into the livers of Dox-fed syngeneic recipients, and, upon tumor manifestation, Dox was removed to induce p53 expression and trigger senescence as above (see Figs. 1 and 2). Regressing tumors were isolated 9 days after Dox withdrawal for 3D imaging of reporter activity and parallel assessment of IFNγ signaling in comparison with proliferating controls (from mice maintained on Dox). As illustrated in Fig. 6C, proliferating tumor cells showed little, if any, reporter expression, whereas tumor cells triggered to senesce *in vivo* displayed a more prominent ZsGreen1 signal (Fig. 6C and D; Supplementary Video S2). This effect coincided with a specific increase in levels of IFNγ protein (but not type I IFN) in tumor tissue extracts (Fig. 6E; Supplementary Fig. S12C).

To test whether the altered composition of immune cells in senescent tumors (Fig. 2; Supplementary Figs. S5–S6) contributed to the enhanced signal of the IGS reporter, we performed *in vitro* coculture assays allowing exposure of senescent or proliferating tumor cells to an equal number of activated CD8 T cells, which we identified via scRNA-seq data as the predominant cellular source of IFNγ *in vivo* (Fig. 6F–H). Senescent cells still showed a significant increase of the ZsGreen1 signal as compared with proliferating controls (Fig. 6I). Consistent with a non–cell autonomous signaling activation, IFNγ was not detected in conditioned media from NSP tumor cells under proliferative or senescent conditions (Supplementary Fig. S12D), yet IFNγ was readily detected upon coculture with CD8 T cells, an effect that was further enhanced by the addition of macrophages and associated with increased MHC class I on senescent cells as well as increased activation of CD8 T cells (Supplementary Fig. S12E–S12J). Collectively, these data support a model whereby heterotypic interactions between senescent tumor cells and immune cells sensitize the tumor to exogenous IFNγ, leading to enhanced antigen presentation and efficient immune surveillance.

### IFNγ Signaling in Senescent Tumor Cells Is Necessary for Immune Surveillance

**Figure 7.**
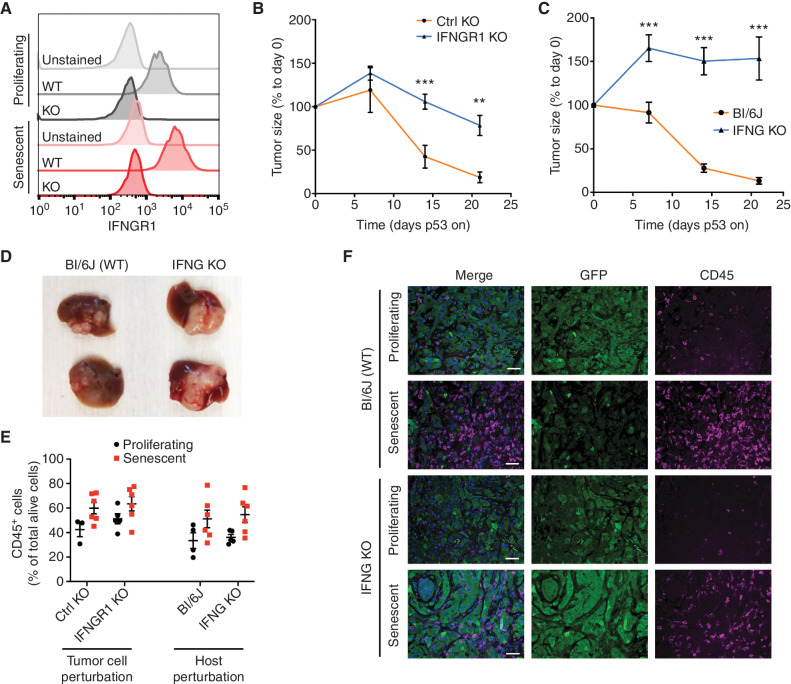
IFNγ signaling in senescent tumor cells is necessary for immune surveillance. **A,***Ifngr1* KO of both proliferating and senescent NSP cells validated by flow cytometry. **B,** Tumor regression phenotype of *Ifngr1* KO or control sgRNA–transfected tumor cells orthotopically injected into Bl/6N mice upon p53 restoration. A control sgRNA targeting a gene desert located on Chr8 (Ctrl KO) serves as a control. **C,** Tumor regression phenotype of parental NSP tumor cells orthotopically injected into WT or *Ifng* KO mice upon p53 restoration. **D,** Representative macroscopic images of tumor collected at day 21 after p53 restoration from **C**. **E,** Flow cytometry analysis of CD45 abundance in tumor from indicated groups. **F,** Representative immunofluorescence in p53-suppressed (proliferating) and p53-restored (senescent, 7 days after p53 restoration) tumor from the indicated host. NSP tumor cells were transduced with GFP-expressing vector for visualization. Scale bars, 50 μm. Data are presented as mean ± SEM. Two-tailed Student *t* test was used. **, *P* < 0.01; ***, *P* < 0.001.

Our results imply that the immune-mediated clearance of senescent NSP tumor cells involves the combined effects of SASP, known to stimulate immune cell recruitment (10, 20, 58), together with a previously underappreciated capacity of senescent cells for enhanced sensing and response to EC signals, as shown here with IFNγ. To test the contribution of the senescence-associated IFNγ sensing program to the immune surveillance of senescent tumor cells, we examined how disruption of the IFNGR in the tumor cells, or IFNγ depletion in the host, affects the clearance of NSP tumor cells upon senescence induction. Indeed, tumor regression (but not senescence per se; Supplementary Fig. S13A–S13D) was impaired upon knockout (KO) of IFNGR1 (Fig. 7A and B; Supplementary Fig. S14A–S14C), an effect that was even more pronounced for IFNGR-intact tumors engrafted into *Ifng^‒/‒^* mice (Fig. 7C and D) and associated with the expected loss of surface MHC-I in tumor cells (Supplementary Fig. S14D and S14E).

Consistent with the known contribution of IFNγ signaling and tumor cell MHC-I to CD8^+^ engagement (59), tumors that lacked IFNGR1 or that developed in *Ifng^‒/‒^* recipients contained fewer CD8 T cells than their WT counterparts in both proliferating and senescent states (Supplementary Fig. S14F) while still inducing robust immune infiltrate including abundant macrophages (Fig. 7E and F; Supplementary Fig. S14G). Regardless, the impaired senescence surveillance phenotype was not simply a result of this decrease in CD8 T cells. Coculture assays providing uniform exposure of IFNGR1 KO and WT tumor cells to CD8 T cells and macrophages still showed IFNGR1-dependent killing of senescent tumor cells (Supplementary Fig. S15A–S15E)—a dependence that required the presence of both T cells and macrophages and that not was observed in proliferating tumor cells under the same conditions. Taken together, these data indicate that enhanced ability of senescent cells to sense microenvironmental IFNγ acts in concert with SASP-stimulated immune cell recruitment to enable mutually reinforcing heterotypic interactions among tumor cells, macrophages, and activated T cells that improve antigen presentation and immune surveillance, leading to potent tumor regressions.

## DISCUSSION

Enabled by a murine tumor model in which cancer immune evasion versus senescence surveillance is under tight genetic control, we reveal how senescent cells dramatically alter their ability to both send and receive environmental signals (Supplementary Fig. S16). Consistent with known senescence programs, p53-driven senescence induction led to the silencing of proliferative genes and induced the SASP. However, we also observed a profound effect on gene expression for PM proteins, including a range of growth factor receptors and cytokine receptors that are predicted to drastically alter how senescent cells respond to environmental signals. Importantly, although we used a liver cancer model as our primary experimental system, a similar rewiring in the expression of cell-surface sensors and gene programs sensitizing to environmental signals was observed in a broad range of murine and human tumor cells treated with senescence-inducing agents, implying that the altered sensing program is a general hallmark of the senescent state.

One of the prominent sensing pathways altered in senescent cells involves type II IFN signaling. In our liver cancer model and across all senescent states we examined, senescence is accompanied by cell-intrinsic transcriptional and protein expression changes predicted to enhance signaling from exogenous IFNγ. Indeed, senescent cells more robustly activated IFNγ effectors in response to IFNγ *in vitro* and *in vivo*, and both an intact IFNγ effector pathway and IFNγ in the environment are required for efficient CD8 T cell–mediated clearance of senescent tumor cells. Although pathway analysis of senescent cell transcriptomes invariably identifies type II IFN signaling as an enriched feature, overlaps between type I and II signaling components and the fact that IFNγ is typically not detected as a SASP factor have left mechanistic questions regarding type II IFN signaling in senescence largely unexplored. Our studies demonstrate that such enrichment in IFN signaling signatures of senescent cells reflects an enhanced capacity for IFNγ sensing whose output is most prominent *in vivo*.

Perhaps the most well-established output of type II IFN signaling involves its ability to induce the antigen-presentation machinery. Indeed, IFNγ induced cell-surface expression of MHC-I (or HLA in human cells) in our model under both proliferating and senescence conditions. However, IFNγ-induced MHC-I upregulation was more pronounced in senescent cells, an effect that correlated with increased expression of the transporter associated with antigen processing, other antigen processing factors, and structural components of MHC-I. A similar hypersensitivity to IFNγ in inducing MHC-I/HLA was observed in human liver and lung cancer cell lines triggered to senesce. These results imply that the senescence program can enhance antigen presentation in non–immune cells, thereby facilitating tumor immunosurveillance.

Our results support a model whereby the ultimate impact of senescent cells on tissue biology is dictated by the combined effects of how they send and receive environmental signals. Not only do senescent cells induce the SASP, which triggers tissue remodeling and alters the cell state and composition of immune cells in the environment, but they also dramatically alter their surfaceome, leading to a differential ability to sense environmental factors, herein exemplified by IFNγ. Importantly, disruption of IFNγ signaling had no effect on senescence induction or the SASP in our system, yet impaired subsequent tumor regressions, indicating that altered environmental sensing acts in concert with the SASP to determine the ultimate output of the senescence program—in this case, immune surveillance. These effects appear to be part of a coordinated epigenetic program, as both the SASP and sensing programs show a prominent dependence on the chromatin remodeling factor BRD4.

Although the mechanism of immune surveillance in our model depends on the cooperative effects of CD8 T-cell and macrophage populations reflective of a transition from an “immune cold” to an “immune hot” tumor microenvironment, other innate or adaptive immune cell types may recognize and clear senescent cells in different contexts, or, alternatively, immune surveillance may not occur at all (18, 19). Undoubtedly, some of these distinctions reflect heterogeneity in SASP factor secretion (13, 14), though our results raise the possibility that the extent and nature of altered environmental sensing may also influence how senescent cells affect tissue biology. Although knockout of IFNγ sensing (via *Ifngr1* KO) and deletion of MHC-I (B2M KO) in senescent tumor cells impaired their immune surveillance *in vivo*, it did not completely abolish tumor regression after senescence induction, indicating that IFNγ sensing in senescent cells is not the only pathway contributing to tumor regression. Regardless, the fact that senescent cells can respond differently to environmental signals implies that their ultimate molecular state in tissues will be different than in cell culture, highlighting the need to better characterize the process *in vivo*.

Our results may help explain the paradoxical effects of senescence biology in physiology and disease and have implications for the effective use of senescence-modulating therapeutics. For example, in our model, the difference between tumor senescent cell clearance and persistence was determined, at least in part, by the presence of environmental IFNγ and the integrity of the type II IFN signaling in the senescent cells. This suggests that variation in the ability of senescent cells to recruit and sense IFNγ-secreting immune cells or other immune cell types could profoundly affect senescent cell clearance, such that decreased environmental IFNγ or diminished type II IFN signaling could enable senescent cell persistence within tissues. In the context of cancer, therapies that induce tumor cell senescence—a cytostatic program—can trigger immune-mediated tumor regression or resensitize tumors to immune-checkpoint blockade, yet these are not the universal outcomes. As such, heterogeneity in the SASP (which can vary between tumor cell types and senescence inducers) or IFNγ sensing and output [perhaps affected by deletion or mutation of IFNγ pathway or HLA components (60) or the reversible transcriptional mechanisms uncovered here] may influence the effectiveness of such therapies in patients. Consistent with this notion, therapy-driven induction of specific SASP profiles predicts patient outcomes in a subgroup of patients with ovarian cancer (61). By contrast, strategies to enhance the immune surveillance of senescent cells by increasing their sensitivity to IFNγ (e.g., with PTPN2 inhibitors) may help bias program output toward tumor cell rejection. We envision that investigating this and other tissue remodeling and sensing programs in pre- and posttreatment tumor biopsies (e.g., through transcriptomic or proteomic profiles) may expose new response biomarkers and/or combination strategies to improve the clinical management of cancer.

## METHODS

### Cell Culture and Drug Treatment

p53-restorable mouse liver cancer cell lines were cultured in DMEM supplemented with 10% FBS and 1% penicillin and streptomycin (Gibco) on plates that were collagen-coated (PurCol, Advanced Biomatrix, 0.1 mg/mL) for 30 minutes at 37°C and maintained by the addition of 1 μg/mL Dox to suppress p53 expression. In order to restore p53 expression and therefore induce senescence, Dox-containing media were replaced with Dox-free media for 6 to 8 days. Cells were replated every 2 to 3 days to wash off Dox. Several cell lines have been generated, and NSP is predominantly used for this study given the robustness of the senescence phenotype upon p53 restoration. For human liver cell lines, HepG2 and SK-Hep1 were cultured with EMEM, and SNU447 was cultured in RPMI 1640 in noncoated, tissue culture–treated plates, all supplemented with 10% FBS and 1% penicillin and streptomycin. For human lung cancer cell lines, A549, H460, and H2030 were cultured in DMEM in noncoated, tissue culture–treated plates supplemented with 10% FBS and 1% penicillin and streptomycin. All human cell lines were obtained from ATCC. Both murine and human cell lines were tested for *Mycoplasma* regularly every 6 months. The concentration and regimen of drug treatment in cancer cell lines were as follows. For perturbing BRD4-dependent transcriptional programs, cells were treated with 500 nmol/L of JQ1 (S7110, Selleckchem) for 48 hours prior to harvest, starting JQ1 at day 6 after restoring p53 (off-Dox), when NSP cells are fully senescent. For drug-induced senescence experiments, p53-suppressed (on-Dox) NSP cells were treated with trametinib (25 nmol/L, S2673 Selleckchem) + palbociclib (500 nmol/L, S1116, Selleckchem), nutlin (10 μmol/L, S1061, Selleckchem), or cisplatin 1 μmol/L), changed every 2 to 3 days, for 7 days. The concentration of DMSO corresponded to the drug treatment and does not exceed 1:1,000 dilution of total media volume, which shows no discernible toxicity to cultured cells. For IFNγ of proliferating or senescent populations, the indicated doses of mouse or human recombinant IFNγ were administrated to murine and human cancer cell lines, respectively, after 24 hours of cell seeding, and cells were harvested after 24 hours of IFNγ treatment for phenotypic or molecular analyses.

### Cell Culture and Drug Treatment

#### Primary Liver Tumor Generation and Isolation of Liver Cell Lines

All mouse experiments were approved by the Memorial Sloan Kettering Cancer Center (MSKCC; New York, NY) Internal Animal Care and Use Committee. Mice were maintained under specific pathogen–free conditions, and food and water were provided *ad libitum*. C57BL/6N female mice ages 8 to 9 weeks old were injected via HTVI with a sterile 2 mL (or 1/10 of mouse body weight) of 0.9% NaCl solution containing 5 μg of pT3-EF1a-NrasG12D-IRES-rtTA (Tet-On system) and 20 μg of pT3-TRE-tRFP-shp53 transposon vectors along with 5 μg CMV-SB13 transposase (5:1 ratio) through the lateral tail vein. Dox was administered to mice via 625 mg/kg Dox-containing food pellets (Harlan Teklad) at least 4 days before the injection. The tumor was harvested 5 to 7 weeks after injection for cell line isolation. To derive cancer cell lines from the primary liver tumor, tumors were minced and digested with 5 mL of digesting solution, containing 1 mg/mL collagenase IV (C5138, Sigma-Aldrich) and 0.3% Dispase II (Roche 04942078001) in DMEM, at 37°C for 30 minutes with occasional vortexing. The cells were spun down to remove the supernatant and plated on a collagen-coated plate. Independent cell lines were passaged at least 7 to 8 passages to remove fibroblasts and obtain a homogenous population. For those experiments involving bioluminescence tracking of tumor growth, the transposon construct pT3-EF1a-NrasG12D-IRES-rtTA-IRES-Luc was used. In the Tet-Off system setting, the transposon construct pT3-EF1a-NrasG12D-IRES-tTA was used to coinject with pT3-TRE-tRFP-shp53 vector into mice under normal diet to allow p53 hairpin expression. To restore p53 in the liver tumor, the mice were subjected to a Dox diet. For the constitutive p53 knockdown model, transposon constructs pT3-EF1a-NrasG12D and pT3-EF1a-tRFP-shp53 were used.

#### Orthotopic Transplant Experiments

Both C57BL/6 mice were predominantly used for the animal study for the HTVI tumor generation and orthotopic liver injection experiments in the immunocompetent setting. The C57BL/6N strain was mainly used except for the matching control strain with IFNG KO mice (The Jackson Laboratory, #002287) that was in the C57BL/6J background. No difference was observed in terms of tumor growth or senescence surveillance phenotype between the C57BL/6N and J strains. Female mice were used in the experiment for the convenience of cage separation. All *in vivo* experiments were performed with age-matched (8–13 weeks old) cohorts. For the orthotopic liver tumor injection, NSP tumor cells were trypsinized and filtered twice using a 40-μm strainer to reduce cell doublets followed by pelleting and were prepared in 20 μL of 1:1 DMEM-to-Matrigel ratio and injected using a 31-gauge needle to the left lobe of the mouse liver following the standard microsurgery institutional practice. Due to the engraftment differences in mice of different strains—C57BL/6, nude, and R2G2 (Envigo) mice—different amounts of tumor cells were injected. Specifically, 5 × 10^5^, 8 × 10^4^, and 5 × 10^4^ cells were injected, respectively in each strain to have comparable tumor size around 2 weeks after injection. Mice were then randomized based on similar size of tumor and assigned to different groups for the subsequent experimental design.

### Lentiviral and Retroviral Production and Transduction

Lentiviruses were generated by cotransfection of viral vectors (1.5 μg) with packaging plasmids psPAX2 (0.75 μg) and pCMV-VSVG (0.25 μg; Addgene) into 293T cells with 90% confluency in a 6-well plate. Retroviruses were generated by cotransfection of viral vectors (2 μg) with pCMV-VSVG (0.25 μg; Addgene) into Phoenix-gp cells with 90% confluency in a 6-well plate. Polyethyleneimine (PEI) was added during cotransfection with a ratio of total DNA:PEI = 1:3 to facilitate the binding of the plasmid to the cell surface. Viral-containing supernatants were cleared of cellular debris by 0.45-μm filtration. Target cells were exposed to viral supernatants and mixed with 4 μg/mL polybrene overnight before being washed, grown for 24 hours in fresh media, and then subjected to antibiotic selection or fluorescence-based cell sorting.

### Lentiviral and Retroviral Vectors

Murine liver cancer cells were infected with the retroviral vector MSCV-Luc2-IRES-GFP (62) to enable bioluminescence imaging. For visualization and staining of liver tumor cells *in vivo*, tumor cells were infected with either the following lentiviral vectors specified in the figure legends, pRRL-SFFV-GFP-mirE(shRen)-PGK-puromycin (SGEP was a gift from Johannes Zuber, Addgene #111170) or pRRL-EFS-GFP-shRen (generated through replacing SFFV with EFS promoter and removing antibiotic selection marker puromycin), to label the cells with GFP. For visualization of IFNγ sensing, tumor cells were infected with the lentiviral IGS reporter construct described below.

### Genetic Manipulation of Cell Line Using CRISPR/Cas9

In order to knock out specific genes in mouse and human liver tumor cell lines, the plasmid pSpCas9(BB)-2A-GFP (PX458; PX458 was a gift from Feng Zhang, Addgene #48138), in which a single-guide RNA (sgRNA) targeting either an intergenic region of chromosome 8 (Ctrl) or the specific gene of interest, was cloned. Cells were transiently transfected with PEI (2 μg plasmid and 6 μL PEI in 6-well plates with 60% confluency). Transfected cells were subsequently FACS sorted by GFP positivity 36 to 48 hours after transfection. For Ifngr1 and B2m KO experiment, PX458-transfected cells were first stained with IFNGR1 (2E2, biotin) followed by streptavidin–APC staining, and MHC-I (H-2k^b^; AF6-88.5.5.3) antibody, respectively, and negative cells were sorted. Sorted populations were further tested with IFNγ to evaluate KO efficiency using MHC-I induction as a proxy. In order to generate p53 KO human tumor cells, cells were electroporated following the manufacturer's instructions. Briefly, cells were trypsinized, washed in PBS once, counted, and then resuspended in Neon Buffer R. In parallel, 1 μg of Cas9 (Thermo Fisher) and 1 μg of sgRNA were complexed for 15 minutes at room temperature to form the Cas9 RNP complex, which was then mixed with the cell aliquot. The cell/RNP mixture was electroporated (1,400 V pulse voltage, 20 ms pulse width, 2 pulses) using a Neon electroporation system (Thermo Fisher). The cells were recovered for 3 days with further selection through nutlin treatment (10 μmol/L; Selleck Chemicals S1061) for 5 to 7 days to enrich p53 KO cells. The sgRNA sequence used in the experiments was as follows: Ifngr1: TGGAGCTTTGACGAGCACTG, B2m: AGTATACTCACGCCACCCAC, Ctrl: GACATTTCTTTCCCCACTGG, and TP53: CGCTATCTGAGCAGCGCTCA.

### Coculture Assays

In order to isolate CD8^+^ T cells from spleens of female OT-I mice (The Jackson Laboratory), spleens were mechanically disrupted by passing them through a 70-μm cell strainer and centrifuged at 1,500 rpm for 5 minutes. Red blood cells were lysed with ACK lysis buffer (Quality Biological) for 5 minutes. Total splenocytes or CD8^+^ T cells FACS sorted on a Sony MA900 were then activated with CD3/CD28 Dynabeads (one bead/T cell; Thermo Fisher) and cultured in the presence of IL2 (2 ng/mL; BioLegend), IL7 (2.5 ng/mL; PeproTech), IL15 (50 ng/mL; PeproTech), and 2-mercaptoethanol (5.5 μmol/L, Fisher Scientific) in complete RPMI 1640 media supplemented with 10% FBS and 100 IU/mL penicillin/streptomycin for 5 to 6 days (passage cells every 2 to 3 days) prior to coculture assays with mouse liver tumor cells. For Kupffer cell isolation, BL/6 male mice ages 8 to 14 weeks were first subjected to liver perfusion as previously described (63). After perfusion, the liver was removed and homogenized and then digested with protease solution (0.5 mg/mL type XIV protease, Sigma, P5147) supplemented with DNase I (0.2 μg/mL, Roche, 10104159001) for 15 minutes at 37°C with constant stirring. This suspension was then centrifuged at 50 × *g* for 3 minutes to remove the hepatocyte pellet. The supernatant was then transferred and centrifuged 580 × *g* for 5 minutes at 4°C. Next, the pellet was washed with HBSS to remove residual protease solution and centrifuged at 580 × *g* for 5 minutes at 4°C to pellet the cells again. The pellet was then resuspended with FACS buffer and subjected to ±-F4/80 isolation according to the manufacturer's instruction (Miltenyi Biotec, 130-110-443). After isolation, the purity of Kupffer cells was confirmed with F4/80 staining through flow cytometry.

Murine liver tumor cells, NSP, were transduced with retrovirus expressing PresentER-SIINFEKL construct (GFP; a gift from David Scheinberg, Addgene #102944) to express the peptide 257-264 from chicken ovalbumin, which is presented by H-2Kb on the cell surface. Transduced cells were further selected with puromycin to obtain > 95% GFP positivity. Tumor cells were cultured in the presence or absence of Dox for 6 days in order to induce senescence. Proliferating (1,000) or senescent (2,000) tumor cells were plated in the individual well of a 96-well collagen-coated plate. For those experiments where Kupffer cells were added, they were isolated on the same day and plated at the indicated ratio 6 hours after plating the tumor cells. Twenty-four hours after plating tumor cells, previously activated OT-I T cells were added at the indicated ratio. Cocultures were imaged over time using an INCell 6000 high-content imager (GE Healthcare Life Sciences), with a 488-nm and a 633-nm laser excitation to visualize tumor cells and T cells (stained by CellTracker Deep Red Dye, Invitrogen C34565), respectively, using a 10× objective. Images were captured at indicated time points, starting after the seeding of T cells onto tumor cell/Kupffer cell cocultures. Images for each channel were saved during the experiment and subsequently analyzed using Columbus image analysis software. GFP^+^ tumor cells were identified and segmented from the background using an intensity-based threshold method. T cells were identified using the same threshold method as the tumor cells. The number of the GFP^+^ tumor cells was quantified and normalized to the untreated control to calculate the killing index.

#### IFNGR1 KO and WT Tumor Cell Mixture in Coculture Experiment

For the IFNGR1 WT versus KO mixture experiment (Supplementary Fig. S15E), cells were mixed and kept on or off Dox for 6 days before starting the coculture experiment using a 24-well plate by plating 7,000 and 14,000 proliferating and senescent cells, respectively, with the same protocol described above. The percentage of IFNGR1 WT versus KO cells and absolute number (through counting beads) were measured by flow cytometry.

#### Effect of Cell–Cell Contact Between Macrophages and CD8 T Cells in Coculture Experiment

To measure the effect of direct contact of macrophages with CD8 T cells, we used a transwell plate (Costar 12 mm transwell, 0.4 μm pore, #3460) to separate macrophage and CD8 T + tumor cells by plating macrophages at the bottom well, whereas the CD8 T cells and tumor cells were plated on the upper well. As a comparison, a 24-well plate with 3 cell types cocultured together was used. After 48 hours, T cells were collected and stained for antibodies and subjected to flow cytometry.

### Proliferation and SA-β-gal Assays

For colony formation assays, 2,500 mouse liver cancer cells or 10,000 human liver cancer cells were plated in each well of a 6-well plate. Cells were cultured for 6 days, fixed with 4% formaldehyde, and stained with crystal violet. Detection of SA-β-gal activity was performed as previously described at pH 5.5 for mouse cells and tissue and pH 6 for human cells (20). For *in vivo* SA-β-gal staining, fresh frozen tissue sections were fixed with 0.5% glutaraldehyde, followed by standard SA-β-gal staining as described above. Sections were counterstained with eosin. For population doubling curves, cells were washed with PBS, trypsinized, and 100,000 cells were plated in triplicates in 6-well plates in the presence or absence of Dox. Every 48 hours, cells were counted and 1 × 10^5^ cells were replated. Population doublings for each 48-hour period were calculated by dividing the final cell number to initial cell number.

### Whole-Mount Immunostaining and Tissue Clearing

To detect T cells and neutrophils in the NSP liver tumors, we performed whole-mount immunostaining and tissue clearing [with benzyl alcohol, benzyl benzoate (BABB)] of excised tumors as previously described (32). At the indicated time points, mice were euthanized by carbon dioxide inhalation and liver tumors were collected and fixed in 4% paraformaldehyde in PBS at 4°C overnight. Tissues were washed three times with PBS for 10 minutes at room temperature and preserved in 0.05% azide in PBS at 4°C before processing. Then, the tissues were permeabilized in methanol (MetOH) gradients in PBS (PBS > 50% MetOH > 80% MetOH > 100% MetOH, 30 minutes in each solution), bleached with Dent's bleach [15% H_2_O_2_, 16.7% dimethyl sulfoxide (DMSO) in MetOH] for 1 hour at room temperature, and rehydrated through descending MetOH gradients in PBS (80% MetOH > 50% MetOH > PBS, 30 minutes in each solution). Tissues were next incubated in blocking buffer (0.3% Triton X-100, 0.2% BSA, 5% DMSO, 0.1% azide, and 25% FBS in PBS) for 24 hours at 4°C on a shaker and then stained with antibodies (rat anti-CD3: clone 17A2, cat. #100202, BioLegend, RRID:AB_312659; goat anti-myeloperoxidase: goatMPO, AF3667, R&D Systems, AB_2250866; and hamster anti-CD31, 2H8, MA3105, Thermo Fisher, RRID:AB_223592, all diluted 1:200 in blocking buffer], for 3 days at 4°C on a shaker. Tissues were next washed for 24 hours in washing buffer (PBS with 0.2% Triton X-100 and 3% NaCl) and stained with secondary antibodies [donkey anti-rat-AF488 (A212008, Invitrogen) and donkey anti-goat AF647 (A21447, Invitrogen) diluted at 1:400 in blocking buffer] for 2 days at 4°C with shaking. Tissues were then washed for 24 hours in washing buffer and thereafter stained with goat anti-hamster-AF568 [goat anti-hamster IgG (H + L) cross-adsorbed secondary antibody, Alexa Fluor 568, A21112, Thermo Fisher, diluted at 1:400] and (1:1,000) in blocking buffer for 2 days at 4°C on a shaker. Tissues were then washed for 24 hours in washing buffer and thereafter dehydrated in MetOH gradients in dH_2_O using glass containers (50% MetOH > 70% MetOH > 90% MetOH > 3 × 100% MetOH, 30 minutes for each step). Tissues were next cleared for 30 minutes in 50% MetOH and 50% BABB (benzyl alcohol, benzyl benzoate, mixed 1:2), followed by clearing 1 hour in 100% BABB. Finally, the tissues were imaged on an SP8 Microscope (Leica). Visualization and quantification were performed with Imaris software (Bitplane). In separate experiments, 3D imaging after tissue clearing was used to detect the ZsGreen1, IGS reporter. For these experiments, we used the CUBIC tissue-clearing protocol that maintains the fluorescence from fluorescent proteins (64). Tissues were excised and fixed as stated above and then were soaked in CUBIC-I solution in a 15-mL conical tube container. CUBIC-I was prepared mixing 108 mL of ddH_2_O with 75 g of urea (Sigma, U5128), 75 g of N,N,N,’N’-Tetrakis(2-Hydroxypropyl)ethylenediamine (Sigma, 122262), and 42 mL of Triton X-100 (Sigma, X100). Samples were maintained at 37°C on a shaker for 7 days, changing the media every other day, until clear. The samples were then counterstained for DAPI in CUBIC-1 (1:1,000) for 24 hours and washed in CUBIC-I overnight. Images were acquired and analyzed as described above.

### Western Blotting

Cells were lysed with RIPA buffer (50 m Tris pH 7.4, 150 mmol/L NaCl, 0.5% sodium deoxycholate, 0.1% SDS; 1 mmol/L EDTA; 1% NP-40) supplemented with phosphatase and protease inhibitor (5872, Cell Signaling Technology) and protein concentration was determined by BCA assay. Samples were boiled for 5 minutes, and 20 to 30 μg of protein was separated by SDS-PAGE, transferred to polyvinylidene difluoride (PVDF) membranes (Millipore) according to standard protocols, and probed with the relevant primary antibody overnight at 4°C. Membranes were then incubated with horseradish peroxidase (HRP)–conjugated anti-rabbit IgG or anti-mouse IgG secondary antibodies (1:10,000, GE Healthcare Life Science) at room temperature, and proteins were detected using Pierce ECL Western Blotting Substrate (34095, Thermo Fisher Scientific). Antibodies were diluted as follows: p53 (CM5; 1:500, NCL-L-p53-CM5p, Leica Biosystems, RRID:AB_2895247), p21 (F-5; 1:500, sc-6246, Santa Cruz Biotechnology, RRID:AB_628073), phospho-STAT1 (Tyr701; 1:500, #9167, Cell Signaling Technology, RRID:AB_561284), phospho-p44/42 MAPK (Erk1/2; 1:1,000, #9101, Cell Signaling Technology, RRID:AB_2315112), STAT1 (1:1,000, #14994, Cell Signaling Technology, RRID:AB_2737027), JAK1 (1:1,000, #3344, Cell Signaling Technology, RRID:AB_2265054), phospho-JAK1 (1:1,000, #3331, Cell Signaling Technology, RRID:AB_2265057), and TC-PTP (PTPN2, 1:1,000, ab180764, Abcam, RRID:AB_2722704). Protein loading was measured using a monoclonal β-ACTIN antibody directly conjugated to HRP (1:20,000; A1978, Sigma-Aldrich, RRID:AB_476692), NUCLEOLIN (1:5,000, ab22758, Abcam, RRID:AB_776878), or VINCULIN (1:2,000, ab129002, Abcam, RRID:AB_11144129). ECL-developed blots were imaged using a FluorChem M system (Protein Simple).

### *In Vitro* Multiplexed ELISA

Conditioned media samples (duplicates collected in complete DMEM 48 hours after seeding) from proliferating or senescent NSP tumor cells (6–8 days after Dox withdrawal) were centrifuged at 1,500 rpm for 3 minutes and filtered through a 0.2-μm filter to remove cell debris. Sample concentrations were normalized by diluting in complete DMEM according to cell count. Aliquots (50 μL) of the conditioned media were analyzed using multiplex immunoassays designed for the mouse (Mouse Cytokine/Chemokine Array 31-Plex) from Eve Technologies. Biological replicates from two independent experiments were performed to determine cytokine levels. Heat maps display relative cytokine expression values normalized to geometric means of individual cytokines from both proliferating and senescent samples.

### Measurement of IFNγ in In Vivo Tumor Lysates and In Vitro Conditioned Medium

BD cytometric bead array, a Mouse Th1/Th2 cytokine kit (cat. # 551287, BD Biosciences) was used to determine IFNγ levels. Flash-frozen tissues were lysed in RIPA buffer and homogenized using TissueLyser II (Qiagen) followed by protein concentration measurement determined by the BCA assay. Tissue lysate (100 μg) was used for subsequent measurement following standard manufacturer instructions of cytometric bead array kits. For *in vitro* conditioned medium measurement, 50 of 200 μL of conditioned medium collected from the 96 well of coculture experiments were used.

### PM-Enriched Mass Spectrometry

To capture differential cell-surface proteome changes induced by senescence, we adapted the protocol from a previously published study (45) and followed the manufacturer's instruction (Pierce Cell-Surface Protein Isolation Kit, #89881) to enrich cell-surface proteins of proliferating and senescent cells through biotin-based labeling, followed by pulldown purification. In brief, we plated one and three 15-cm plates of proliferating and senescent cells (6 days after Dox withdrawal) with an initial seeding of 7 × 10^5^ and 2 × 10^6^ million cells, respectively, and collected the cells 2 days later, with the cells approximatively at 85% confluency. Before the cells were harvested, they were incubated with biotin solution for 30 minutes at 4°C to allow the surface protein labeling. Cells were then washed with cold PBS and scraped down, followed by lysis (buffer provided in the kit). Lysates were centrifuged, and the clarified supernatant was used for the purification of biotinylated proteins on NeutrAvidin Agarose. The supernatant was incubated with NeutrAvidin Agarose for 2 hours at room temperature in the closed column to allow biotinylated protein binding. The column containing Agarose slurry was washed to remove unbound proteins. The proteins were then digested *in situ* in the column overnight using 4 μg of trypsin (Promega, V5111) per column at 37°C on a rotor. Digested proteins were further desalted by C18 Stagetip and subjected to liquid chromatography–mass spectrometry (LC-MS/MS) followed by protein identification through Proteome Discover (Thermo Scientific) according to protocols previously described (45). Nonbiotinylated cell lysates were also included and served as background controls.

### Protein Identification

The LC-MS/MS .raw files were processed using Mascot and searched for protein identification against the SwissProt protein database for human/mouse (please adjust the species accordingly). Carbamidomethylation of C was set as a fixed modification, and the following variable modifications were allowed: oxidation (M), N-terminal protein acetylation, deamidation (N and Q), and phosphorylation (S, T, and Y). Search parameters specified an MS tolerance of 10 ppm, an MS/MS tolerance at 0.080 Da, and full trypsin digestion, allowing for up to two missed cleavages. The FDR was restricted to 1% in both protein and peptide levels. Normalized protein intensities were obtained using Scaffold (4.8.4).

### RNA Preparation and High-throughput RNA-seq Analysis

For *in vitro* liver cell line RNA preparation, total RNA was extracted using TRIzol (Thermo Fisher Scientific) following the manufacturer's instructions. For *in vivo* bulk tumor RNA-seq, proliferating tumor (p53 off) was harvested 7 to 10 days after the randomization point and senescent-induced tumor (p53 on) was harvested 12 days after p53 restoration, allowing a similar size of tumor at harvest. To extract tissue RNA, freshly isolated tumor chunk was first stored in RNA-later solution (AM7024, Thermo Scientific) to preserve RNA integrity until extraction and an RNeasy kit (74106, Qiagen) was used to purify tissue RNA following the manufacturer's instructions. Purified polyA mRNA was subsequently fragmented, and first- and second-strand cDNA synthesis was performed using standard Illumina mRNA TruSeq library preparation protocols. Double-stranded cDNA was subsequently processed for TruSeq dual-index Illumina library generation. For sequencing, pooled multiplexed libraries were run on a HiSeq 2500 machine on RAPID mode. Approximately 10 million 76-bp, single-end reads were retrieved per replicate condition. Resulting RNA-seq data were analyzed by removing adapter sequences using Trimmomatic (65), aligning sequencing data to GRCm38—mm10 with STAR (66), and quantifying genome-wide transcript count using featureCounts (67) to generate raw count matrix. Differential gene expression analysis was performed using the DESeq2 package (68) between experimental conditions, using 3 independent biological replicates (independent cultures of NSP tumor cells) per condition, implemented in R (http://cran.r-project.org/). DEGs were determined by > 2-fold change in gene expression with adjusted *P* < 0.05. For heat map visualization of DEGs, samples were z-score normalized and plotted using “pheatmap” package in R. Functional enrichments of these DEGs were performed with the enrichment analysis tool Enrichr (69). Gene expressions of RNA-seq data were clustered using hierarchical clustering based on the one minus Pearson correlation test. Subtype-specific gene signatures were derived (22) and used as inputs for signature score calculation using the R package singscore (70).

### Public Dataset Transcriptomic Analyses

The signature of different human liver cancer subtypes was obtained from a previous study (22). In brief, the top 200 overexpressed and underexpressed gene transcripts among each tumor subtype were selected as their signature. To analyze the transcriptomic changes of genes encoding PM and EC factors distinguishing senescent and proliferating tumor cells, transcriptomic data of a series of human tumor cell lines triggered to senesce was used according to the previously published study (44) and obtained from the website https://ccb.nki.nl/publications/cancer-senescence/. The expression of selected genes was compared between senescent and the corresponding proliferating cells among individual cell lines and normalized to determine the fold change. Information about protein subcellular localization was derived from the Compartments_knowledge_based database (71), with the genes assigned to specific subcellular localization when the criteria score is e3. The Cancer Genome Atlas Liver Hepatocellular Carcinoma (TCGA-LIHC) dataset, including p53 mutational status, transcriptomic profiles, and patient survival, was downloaded using the R package TCGAbiolinks (72, 73). Senescence signatures derived from our mouse models were used as input for computing signature scores using ssgsea method in the R package GSVA (74). These signature scores were used to separate patients into high and low groups, and the log-rank test was used to test the differences in survival between these two groups.

### Gene Set Enrichment Analysis

Gene set enrichment analysis (GSEA) was performed using the GSEAPreranked tool for conducting GSEA of data derived from RNA-seq experiments (version 2.07) against signatures in the Molecular Signatures Database (MSigDB; http://software.broadinstitute.org/gsea/msigdb), signatures derived herein, and published expression signatures in organoid models and human samples. The metric scores were calculated using the sign of the fold change multiplied by the inverse of the *P* value.

### Reverse Transcription and Quantitative PCR

Total RNA was isolated from the mouse liver tumor cell line using TRIzol (Thermo Fisher Scientific) following the manufacturer's instructions. cDNA was obtained from 500 ng RNA using the Transcriptor First Strand cDNA Synthesis Kit (04896866001, Roche) after treatment with DNase I (18068015, Thermo Fisher Scientific) following the manufacturer's instructions using random hexamer method. The following primer sets for mouse sequences were used: Tap1_F 52-GGACTTGCCTTGTTCCGAGAG-32, Tap1_R 52-GCTGCCACATAACTGATAGCGA-32, Psmb8_F 52-ATGGCGTTACTGGATCTGTGC-32, Psmb8_R 52-CGCGGAGAAACTGTAGTGTCC-32, Nlrc5_F 52-CCTGCGTCCCAGTCATTC-32, Nlrc5_R 52-CTGCTGGTCAGTGATGGAGA-32, Erap1_F 52-TAATGGAGACTCATTCCCTTGGA-32, Erap1_R 52-AAAGTCAGAGTGCTGAGGTTT G-32, H2-K1_F 52-GCTGGTGAAGCAGAGAGACTCAG-32, H2-K1_R 52-GGTGACTTTATCTTC AGGTCTGCT-32, H2-D1_F 52-AGTGGTGCTGCAGAGCATTACAA-32, H2-D1_R 52-GGTGAC TTCACCTTTAGATCTGGG-32, B2m_F 52-TTCTGGTGCTTGTCTCACTGA-32, B2m_R 52-CAG TATGTTCGGCTTCCCATTC-32, Cdkn1a_F 52-CGGTGTCAGAGTCTAGGGGA-32, Cdkn1a_R ATC ACCAGGATTGGACATGG-32, Trp53_F 52-CTAGCATTCAGGCCCTCATC-32, Trp53_R 52-TCCGACTGTGACTCCTCCAT-32, Csf3_F 52-ATGGCTCAACTTTCTGCCCAG-32, Csf3_R 52- CTGACAGTGACCAGGGGAAC-32, Socs3_F 52-ATGGTCACCCACAGCAAGTTT-32, Socs3_R 52-TCCAGTAGAATCCGCTCTCCT-32, Ptpn2_F 52-ATGTCGGCAACCATCGAGC-32, Ptpn2_R 52-TGTTTCGGTTTCTGTTTTCTGGA-32, Irf1_F 52-ATGCCAATCACTCGAATGCG-32, Irf1_R 52-TTGTATCGGCCTGTGTGAATG-32, Ccl5_F 52-CTGCTGCTTTGCCTACCTCT-32, Ccl5_R 52-CGAGTGACAAACACGACTGC-32, Il18-F 52-CAGGCCTGACATCTTCTGCAA-32, Il18-R 52-TCTGACATGGCAGCCATTGT-32, Hprt_F 52-TCAGTCAACGGGGGACATAAA-32, Hprt_R 52-GGGGCTGTACTGCTTAACCAG-32, Rplp0_F 52-GCTCCAAGCAGATGCAGCA-32, and Rplp0_R 52-CCGGATGTGAGGCAGCAG-32. Quantitative PCR with reverse transcription (qRT-PCR) was carried out in triplicate (10 cDNA ng per reaction) using SYBR Green PCR Master Mix (Applied Biosystems) on the ViiA 7 Real-Time PCR System (Life Technologies). Hprt and Rplp0 (also known as 36b4) served as endogenous normalization controls.

### Tumor Measurement by Ultrasound and Bioluminescence Imaging

High-contrast ultrasound imaging was performed on a Vevo 2100 System with an MS250 13- to 24-MHz scan head (VisualSonics) to stage and quantify liver tumor burden. Tumor volume was analyzed using Vevo LAB software. Bioluminescence imaging was used to track luciferase expression in orthotopically injected liver tumor cells expressing a Luc-GFP reporter as well as primary HTVI tumor harboring luciferase construct (vector described above). Mice were injected i.p. with luciferin (5 mg/mouse; Gold Technologies) and then imaged on a Xenogen IVIS Spectrum imager (PerkinElmer) 10 minutes later. Quantification of luciferase signaling was analyzed using Living Image software (Caliper Life Sciences).

### Flow Cytometry and Sample Preparation

For *in vivo* sample preparation, orthotopically injected liver tumors were isolated by removing the adjacent normal tissue and allocated for 10% formalin fixation, OCT frozen blocks, snap-frozen tissue, and flow cytometry analysis. To prepare single-cell suspensions for flow cytometry analysis, the liver tumor was mechanically disrupted to a single-cell suspension using a 150-μm metal mesh and glass pestle in ice-cold 3% FBS/HBSS and passed through a 70-μm strainer. The liver homogenate was spun down at 400 × *g* for 5 minutes at 4°C, and the pellet was resuspended in 15 mL 3% FCS/HBSS, 500 μL (500 U) heparin, and 8 mL Percoll (GE), mixed by inversion, and spun at 500 × *g* for 10 minutes at 4°C. After the removal of the supernatant, cells were resuspended in PBS supplemented with 2% FBS. Samples were blocked with anti-CD16/32 (1:200, FC block, #553142; BD Pharmingen) for 20 minutes and then incubated with the following antibodies for 30 minutes on ice: CD3 (1:200, 17A2, #612803, RRID:AB_2870130), CD19 (1:200, 1D3, #563235, RRID:AB_2738085), CD4 (1:800, RM4-5, #563151, RRID:AB_2687549), Ly6G (1:200, 1A8, #563005, RRID:AB_2737946), CD44 (1:200, IM7, #560568, RRID:AB_1727481), CD11b (1:800, M1/70, #563553, RRID:AB_2738276; BD Biosciences); MHC-I (1:100, H-2k^b^; AF6-88.5.5.3, #17-5958-82, RRID:AB_1311280), CD119 (1:100, 2E2, #13-1191-82, RRID:AB_2572773), Armenian Hamster IgG isotype (1:100, eBio299Arm, #13488881, RRID:AB_470094; Thermo Fisher); CD45 (1:400, 30-F11M, #103128, RRID:AB_493715), Gr-1 (1:200, RB6-8C5, #108406, RRID:AB_313371), F4/80 (1:100, BM8, #123116, RRID:AB_893481), CD8 (1:400, 53-6.7, #100721, RRID:AB_312760), Ly6C (1:200, HK1.4, #128026, RRID:AB_10640120), CD11c (1:200, N418, #117335, RRID:AB_11219204), CD69 (1:200, H1.2F3, #104522, RRID:AB_2260065), CD106 (1:100, MVCAM.A, #105717, RRID:AB_1877142), CD62L (1:200, MEL-14, #104435, RRID:AB_10900082), PD-1 (1:100, 29F.1A12, #135215, RRID:AB_10696422; BioLegend); IFNGR2 (1:100, REA381, #130-105-670, RRID:AB_2652258; Miltenyi Biotec); streptavidin (1:200, #20-4317-U100), TIGIT (1:100, 1G9, #20-1421-U025, RRID:AB_2621591), NK1.1 (1:100, PK136, #65-5941-U100, RRID:AB_2621910; Tonbo); and human antibody HLA-A, B, C (1:100, W6/32, #17-9983-42, RRID:AB_10733389; Thermo Fisher). To distinguish live/dead cells, DAPI and Ghost dye violet 510 (1:1,000, #13-0870-T100; Tonbo) were used depending on whether the cells were fixed. For fixed cells, cells were stained in PBS prior to antibody staining. Flow cytometry was performed on an LSRFortessa or Guava flow cytometer (Luminex Corporation), and data were analyzed using FlowJo (TreeStar).

### Neutralizing Antibody and Liposomal Clodronate Studies

To determine the specific immune cell dependency of senescence surveillance, depleting antibodies or drugs were administrated to the mice 1 day after Dox withdrawal. For NK-cell depletion, mice were injected i.p. with an ±-NK1.1 antibody (250 μg; PK136, Bio X Cell) twice per week. For T-cell depletion, mice were injected i.p. with either an ±-CD4 (200 μg; GK1.5, Bio X Cell) or ±-CD8 antibody (200 μg; 2.43, Bio X Cell) twice per week. Depletion of NK cells, CD4^+^ T cells, and CD8^+^ T cells was confirmed by flow-cytometric analysis of liver tumor tissue. For neutrophil/myeloid-derived suppressive cell depletion, mice were injected intraperitoneally with an ±-Gr-1 (200 μg; RB6-8C5, Bio X Cell) twice per week. For control, the isotype control antibody (200 μg; LTF-2, Bio X Cell) was injected i.p. twice per week. For macrophage depletion, mice were injected i.v. with clodronate liposomes (50 mg/kg of mouse weight; ClodronateLiposomes.com) twice per week. PBS was used as a control.

### Immunofluorescence and IHC

Tissues were fixed overnight in 10% neutral buffered formalin (Richard-Allan Scientific), embedded in paraffin, and cut into 5-μm sections. Sections were deparaffinized and rehydrated with a histoclear/alcohol series and subjected to antigen retrieval by boiling in citrate antigen retrieval buffer (Vector). Slides were then blocked in PBS/0.1% Triton X-100 containing 1% BSA. Primary antibodies were incubated overnight at 4°C in a blocking buffer. The following primary antibodies were used: GFP (ab13970, Abcam, 1:500, RRID:AB_300798), Ki-67 (#550609, BD Biosciences, 1:200, RRID:AB_393778), CD8 (#14-0808-82, eBioscience, 1:200, RRID:AB_2572861), CD45 (#70257, Cell Signaling Technology, 1:100, RRID:AB_2799780), F4/80 (#70076, Cell Signaling Technology, 1:200), and p21 (#556431, BD Biosciences, 1:200, RRID:AB_396415). For IHC, Vector ImmPress HRP kits and ImmPact DAB (Vector Laboratories) were used for secondary detection. For immunofluorescence, the following secondary antibodies were used: goat anti-chicken AF488 (A11039, Invitrogen, 1:500, RRID:AB_2534096), donkey anti-rabbit AF594 (A21207, Invitrogen, 1:500, RRID:AB_141637), goat anti-rabbit AF594 (A11037, Invitrogen, 1:500, RRID:AB_2534095), and donkey anti-rabbit AF647 (A31573, Invitrogen, 1:500, RRID:AB_2536183). All secondary antibodies were diluted in a blocking buffer and incubated for 1 hour at room temperature. Subsequently, slides were washed, and nuclei were counterstained with PBS containing DAPI (1 μg/mL) and mounted under coverslips with ProLong Gold (Life Technologies). Images were acquired with a Zeiss AxioImager microscope using Axiovision software.

Triple immunofluorescence staining of PD-L1, CD68, and GFP of 2- to 3- μm sections was performed using a Leica Bond RX platform (Leica Biosystems) with ER2 buffer (AR9640, Leica Biosystems) for epitope retrieval. The following primary antibodies were used: PD-L1 (D5V3B, #64988, Cell Signaling Technology, 1:150, 30 minutes, RRID:AB_2799672), CD68 (orb47985, Biorbyt, 1:1,000, 30 minutes), and GFP (TP401, Amsbio, 1:1,000, overnight, after Opal protocol, RRID:AB_10890443). Antibodies were detected using an Opal 4-Color Automation IHC Kit (NEL8720001KT, Akoya) with Opal 520 Reagent for PD-L1, Opal 570 Reagent for CD68, and BrightVision Poly-HRP-Anti Rabbit antibody (DPVR-110HRP, Immunologic). GFP was visualized using donkey anti-rabbit IgG (H + L) Alexa Fluor 647 (A31573, Thermo Fisher, 1:500, 1 hour, RRID:AB_2536183). Nuclei were stained with DAPI (D9542, Sigma). The specificity of staining was confirmed with polyclonal rabbit IgG (Abcam, ab37415, RRID:AB_2631996). Fluorescence images were acquired with a NanoZoomer-2.0 HT C9600 digital scanner (Hamamatsu) and visualized with QuPath software (75) using the same settings.

### Generation of the IGS Reporter

In order to generate the IGS reporter from our study, we have adapted the construct design from the previously described article (57). In brief, we have crafted a 5× Interferon Gamma-activated sequence inserted in front of a mini promoter (minimal TATA-box promoter with low basal activity) followed by a ZsGreen1 reporter. Right after the reporter sequence, this lentiviral construct also contains RFP driven by the PGK promoter to have constitutive RFP expression for cell visualization. The cells were transduced with virus and sorted through flow cytometry with high RFP level for stable expression of the construct in the cells.

### scRNA-seq Analyses

#### Data Preprocessing and Quality Control

All scRNA-seq data were processed into count matrices using 10X Genomics CellRanger 6.0.0 with default parameters using reference mouse genome GRCm38/mm10 augmented with BioLegend TotalSeqB hashtag oligonucleotide barcode sequences for demultiplexing cellular compartments in downstream analysis. Count matrices were processed to remove empty and low-quality droplets by removing (in order) transcripts with more than 10 million or fewer than 100 total reads, droplets with library size greater than 150,000 or lower than 300 total read count, and droplets with fewer than 15 distinct expressed transcripts.

Potential doublets were removed using Solo version 1.2 (76) by training one doublet classification model per sample using default parameters for Solo and removing droplets using a threshold of 0.5. Subsequently, dead/dying cells were filtered by removing droplets with high mitochondrial RNA content (greater than 20% of transcript counts mapped to mitochondrial genes) or high ribosomal transcript count (greater than 15% of total transcript counts mapped to ribosomal genes). After preprocessing, poor quality samples with either low numbers of recovered cells or a low number of distinct transcripts recovered were removed.

#### scRNA-seq Normalization and Dimensionality Reduction

Filtered count matrices for each sample were combined into a single dataset, normalized to counts per million, and log-transformed. Unwanted variation due to total transcript counts and percentage of mitochondrial reads per cell were regressed out prior to scaling each transcript to zero mean and unit variance. Principal component analysis (PCA) was then performed on a restricted subset of 5,000 highly variable genes using the method described in ref. 77 and implemented in Scanpy version 1.8.2. The top 50 principal components were kept to create a uniform manifold approximation and projection (UMAP; ref. 78) using k = 10 nearest neighbors to obtain a nonlinear 2D embedding for downstream visualization. Leiden clustering (79) was implemented using Scanpy version 1.8.2.

The entropy was computed per collection date for each Leiden cluster and revealed 4/42 clusters with more than 100 cells had low batch entropy (<0.5). Harmony integration was therefore applied for batch correction (80) using 40 PCs. Reclustered cells verified that after integration, all Leiden clusters with more than 100 cells had batch entropy > 0.5.

#### scRNA-seq Compartment Demultiplexing and Cell-type Assignment

Cellular compartments were identified from postintegration Leiden clusters using HashSolo (76). To specifically identify immune cells, we first used hashtag oligonucleotide barcodes matching FACS-sorted CD45^+^ populations, and all cells clustering with this compartment were included in the majority label. We manually validated the compartment calls using canonical markers (i.e., *Ptprc*/CD45 positive; *Vim*, *Col1a2*, and *Krt8* negative, for immune cells). A final count of 13,236 (6,664 from senescent tumors and 6,572 from proliferating tumors) CD45 cells and 17,782 genes across 5 samples were recovered and further analyzed using the workflow below.

To define immune subtype clusters, PCA, UMAP (with 10 PCs and 10 neighbors), and Leiden clustering at low resolution (k = 10, resolution = 0.5) were repeated in immune compartment cells. Major immune subtypes were annotated by examining top DEGs in conjunction with the following marker genes: T cells and NK cells (*Cd3e* and *Nkg7*), B cells (*Cd19*), plasma cells (*Jchain*), granulocytes (*Cxcr2* and *Csf3r*), dendritic cells (*Clec9a*), monocytes/macrophages (*Cd68, Mafb*, and *Csf1r*), and basophils (*Il3ra* and *Cxcr2*).

DEGs were computed by contrasting each Leiden cluster against all other immune cells using the “rank_gene_groups” function implemented in Scanpy version 1.8.2 using the Wilcoxon rank sum test with Benjamini–Hochberg correction.

This annotation process was repeated on the T/NK-cell subset to characterize the following phenotypes by marker gene expression: naïve CD4 T cells (*Cd4^+^ Foxp3^‒^ Cd69^‒^*), activated CD4 T cells (*Cd4^+^ Cd69^+^*), CD4^+^ Tregs (*Cd4^+^ Foxp3^+^*), naïve CD8 T cells (*Cd8^+^ Cd69^‒^ Lag3^‒^*, senescent-enriched CD8 T cells (*Cd8^+^ Cd69^+^*), proliferating-enriched CD8 T cells (*Cd8^+^ Lag3^+^*), and NKT cells (*Klrb1c^+^*).

#### scRNA-seq Analysis of T/NK Cells

Differential abundance analysis using Milo was performed specifically on T-cell subsets (35) using a neighborhood size of *n* = 50. Differentially abundant T-cell subtypes were identified by applying the Simes method for multiple hypothesis correction within a subtype cluster or Benjamini–Hochberg FDR correction across subtypes (setting FDR threshold at 0.2). Enrichment of a subset of *Cd8*^+^*Cd69*^+^ T cells upon p53 reactivation was denoted “senescent-enriched CD8 T cells” and depletion of a subset of Cd8^+^ Cd69^+^ T cells was denoted “growing-enriched CD8 T cells.” For visualization purposes, significantly differentially enriched/depleted neighborhoods by SpatialFDR were visualized as implemented in the MiloPy Python package at an FDR threshold of 0.2.

Differential gene expression analysis within CD8^+^ T-cell compartment was performed using the “rank_gene_groups” function as implemented in Python package Scanpy version 1.8.2. GSEA using GSEA prerank as implemented in Python package GSEApy version 0.12.1 was performed using genes ranked by logFC. Genes were denoted as differentially expressed using an adjusted *P*-value cutoff of 0.05, and any human gene sets tested were mapped to corresponding mouse orthologs using Ensembl annotations.

#### scRNA-seq Analysis of Monocyte/Macrophages

Similar to the T/NK-cell analysis, unbiased differential abundance analysis was performed using Milo as described above, but with a larger neighborhood size of 500 to account for the increase in cell number in the monocyte/macrophage cluster as well as aggregation at the level of Leiden clusters, not marker-defined subtypes.

Two Leiden clusters, one significantly enriched and one depleted upon p53 reactivation, were isolated for differential expression analysis and gene set enrichment as described in the preceding section, and significantly enriched/depleted neighborhoods were visualized using the same SpatialFDR cutoff of 0.2.

### Statistical Analyses

Statistical analyses were performed as described in the figure legend for each experiment. Group size was determined on the basis of the results of preliminary experiments, and no statistical method was used to predetermine sample size. The indicated sample size (*n*) represents biological replicates. All samples that met proper experimental conditions were included in the analysis. In particular, we have observed that in the orthotopic transplantation setting, the undesired lung metastasis (lung weight > 300 mg) occurred due to the technical limitation of liver injection. The lung metastasis may affect the tumor regression phenotype upon p53 restoration, and the mice were thus excluded from the analysis. Survival was measured using the Kaplan–Meier method. Statistical significance was determined by the Student *t* test, log-rank test, Mann–Whitney test, Fisher exact test, and Pearson correlation using Prism 6 Software (GraphPad Software) as indicated. Significance was set at *P* < 0.05.

### Figure Preparation

Figures were prepared using BioRender.com for scientific illustrations and Illustrator CC 2020 (Adobe).

### Data Availability

Bulk and single-cell RNA-seq data generated in this study are available in the Gene Expression Omnibus database under the super-series GSE203140. Code for scRNA-seq data analysis is available at https://github.com/calico/Senescence_CD45. The mass spectrometry proteomics data have been deposited to the ProteomeXchange Consortium via the PRIDE partner repository with the dataset identifier PXD034465.

## Supplementary Material

Supplementary Table S1 Differential expression analyses of CD8 T and macrophages populations of proliferating (p53 off) vs. senescent (p53 on) tumors by scRNA-seq.

Supplementary Table S2 RNA-Seq data of proliferating (PRO) or senescent (SEN) NSP liver tumor cells, for both p53-restoration and drug-induced (trametinib+palbociclib) settings. PRO and SEN cells were also treated with the BET inhibitor JQ-1 (500 n, 48 h), to expose BRD4-mediated transcriptional output in each cellular state.

Supplementary Table S3 Low (50 pg/ml) and high (1 ng/ml) dose of IFN-γ treatment in proliferating and senescent NSP cells.

Supplementary Figure S1-S16 Supplementary figures complement main figures to show that senescent cells have a rewired environmental signal sensing phenotype, exemplified by an enhanced IFN-g signaling, to facilitate anti-tumor immunity.

Supplementary Video S1

Supplementary Video S2
