# Supplementary material for: Senescence Rewires Microenvironment Sensing to Facilitate Antitumor Immunity
Source: Cancer Discov. 2022 Oct 27;13(2):432–53. doi: 10.1158/2159-8290.CD-22-0528 (PMC9901536; doi:10.1158/2159-8290.CD-22-0528)
Supplement: Supplementary Figure S1-S16 — Supplementary figures complement main figures to show that senescent cells have a rewired environmental signal sensing phenotype, exemplified by an enhanced IFN-g signaling, to facilitate anti-tumor immunity. [file cd-22-0528_supplementary_figure_s1-s16_suppsf1.pdf]

Supplementary Fig. S1. The p53-restorable liver cancer model resembles aggressive´Proliferation class´ of human HCC.

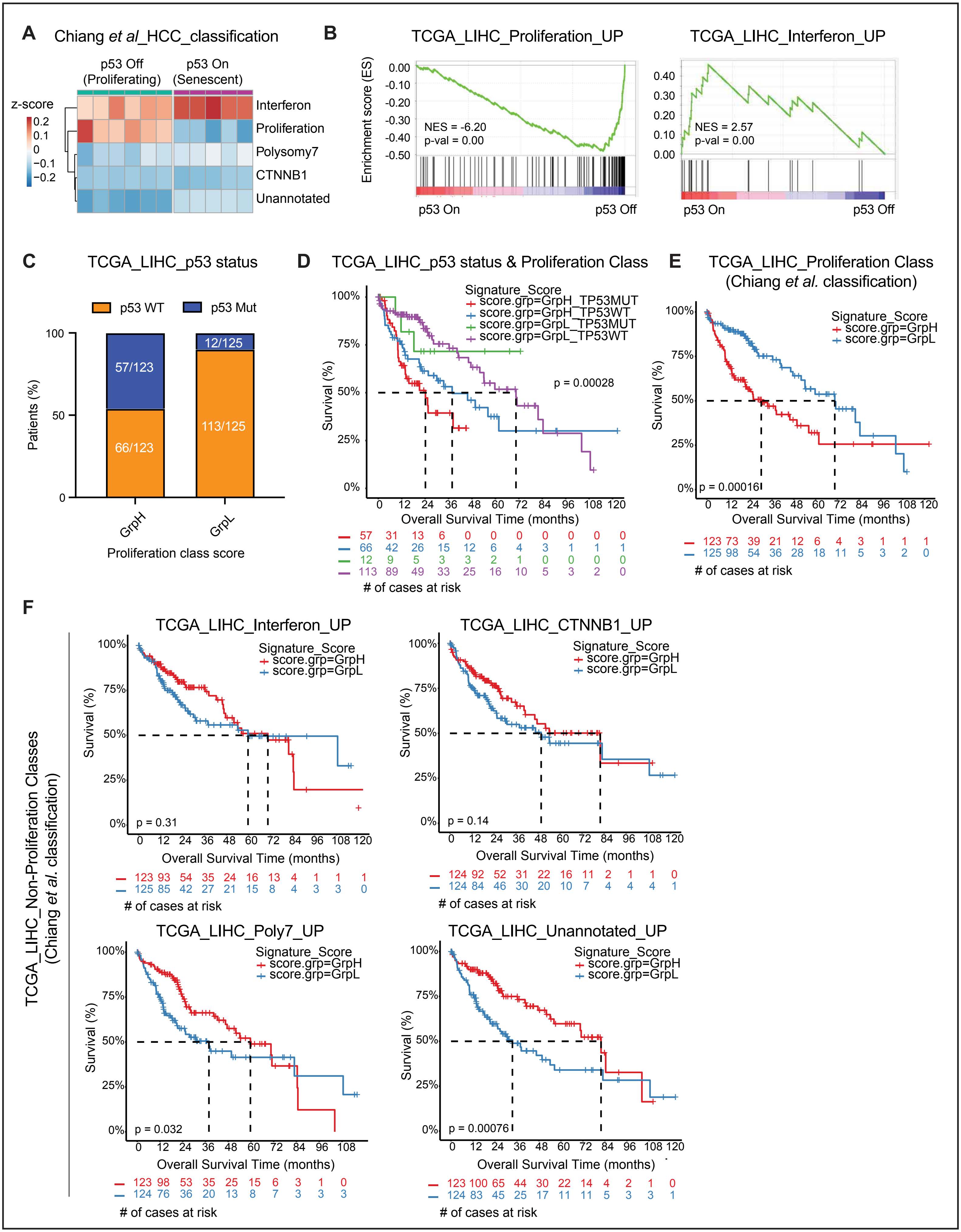

Supplementary Fig. S1. The p53-restorable liver cancer model resembles the aggressive ´Proliferation class´ of human hepatocellular carcinoma (HCC)

A, Classification of p53-restorable HTVI liver tumors using bulk RNA-seq using signatures from publicly available dataset. p53 On tumors were collected at 12 days after Dox withdrawal.

B, Gene Set Enrichment Analysis (GSEA) between p53 On and p53 Off HTVI liver tumor using gene signatures defining human HCC from the Proliferation Class (left) or Interferon Class (right). p53 restoration blunts the expression "proliferation class"-defining genes, enhancing gene programs characteristic of the immunogenic interferon class (21).

C and D, TCGA data of liver cancer patients grouped by Proliferation Class signature expression (top 33% [GrpH] and bottom 33% [GrpL]) and further stratified by *TP53* mutation status (C). Patients with both high score in "Proliferation signature" and p53 mutation, both recapitulated in the p53-restorable HTVI liver tumor model, experience the worst prognosis (D).

E and F, Kaplan-Meier plot of TCGA liver cancer patients stratified by different HCC subclasses signatures. The classification can be divided into two major groups, "Proliferation class" and "non-Proliferation class", and the latter consists of 4 subgroups including "CTNNB1", "Poly7", "Interferon" and "Unannotated".

Supplementary Fig. S2. Kinetics of senescence establishment in NSP tumor cells from p53-restorable liver cancer model

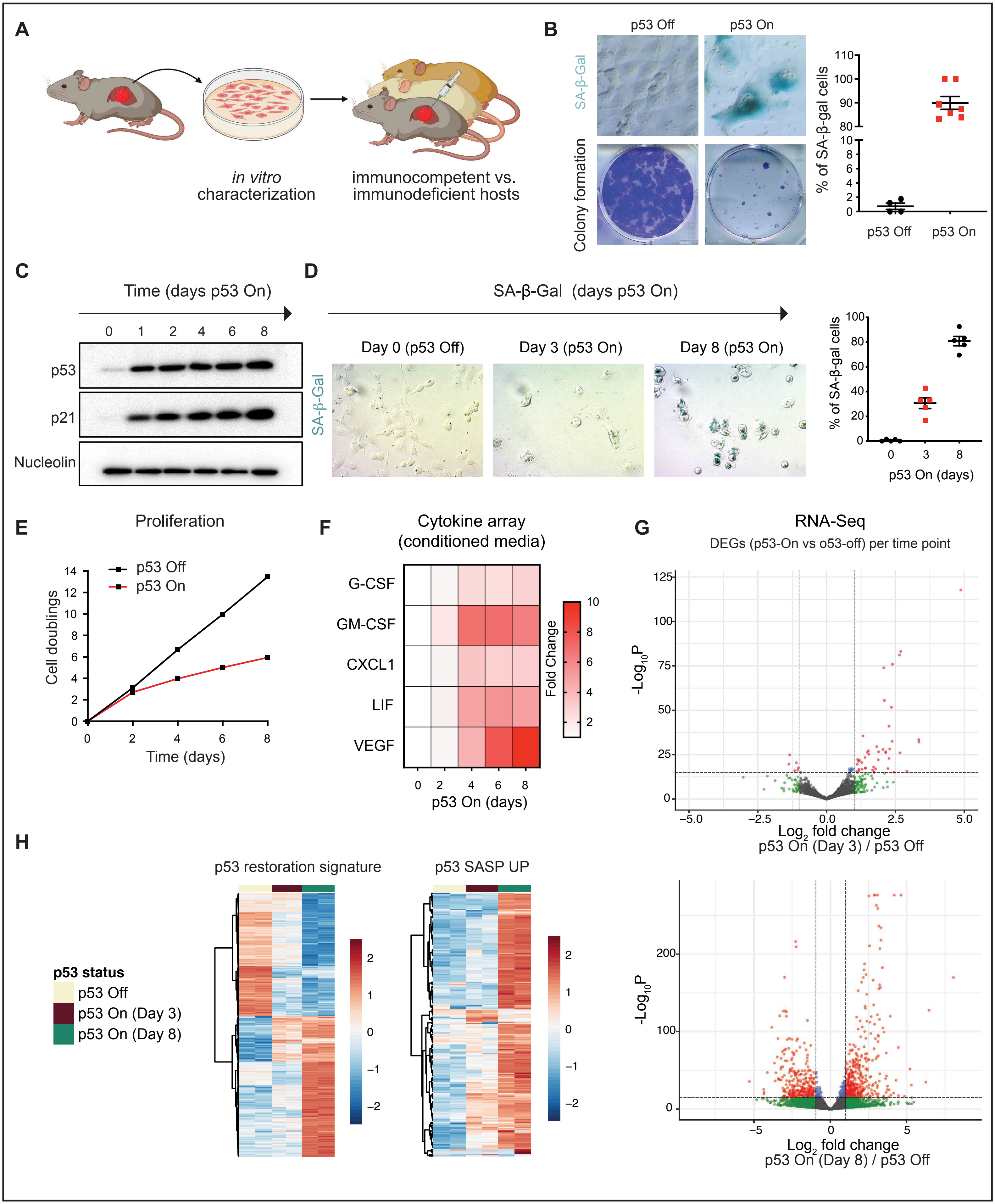

Supplementary Fig. S2. Kinetics of senescence establishment in NSP tumor cells from p53-restorable liver cancer model

A, Graphic illustration of cell lines generation from the HTVI model and subsequent *in vivo* tumor cells orthotopic injection experiment. (Created with BioRender.com.)

B, *In vitro* characterization of p53-restorable liver cancer cell line, NSP, by colony formation assay and SA-β-Gal staining. Several lines have been generated and NSP line is predominantly used for the *in vitro* and orthotopic injection study.

C, Immunoblot analysis of p53-restorable tumor cells cultured *in vitro*.

D, SA-β-Gal staining of NSP cells at the indicated time points after p53 restoration.

E, Comparison of cell doublings in p53 On and p53 Off tumor cells. Experiment is performed in triplicate wells.

F, Cytokine array of conditioned medium collected at the indicated time points after p53 restoration. Cytokine level was normalized to p53 On Day 0 sample.

G and H, RNA-seq analysis of NSP cells with p53 off or p53 on for 3 and 8 days. p53 restoration signature and p53 SASP UP signature are derived from p53 On 8 days NSP cells.

Supplementary Fig. S3. Characterization of senescence-induced tumor regression in NSP transplant model

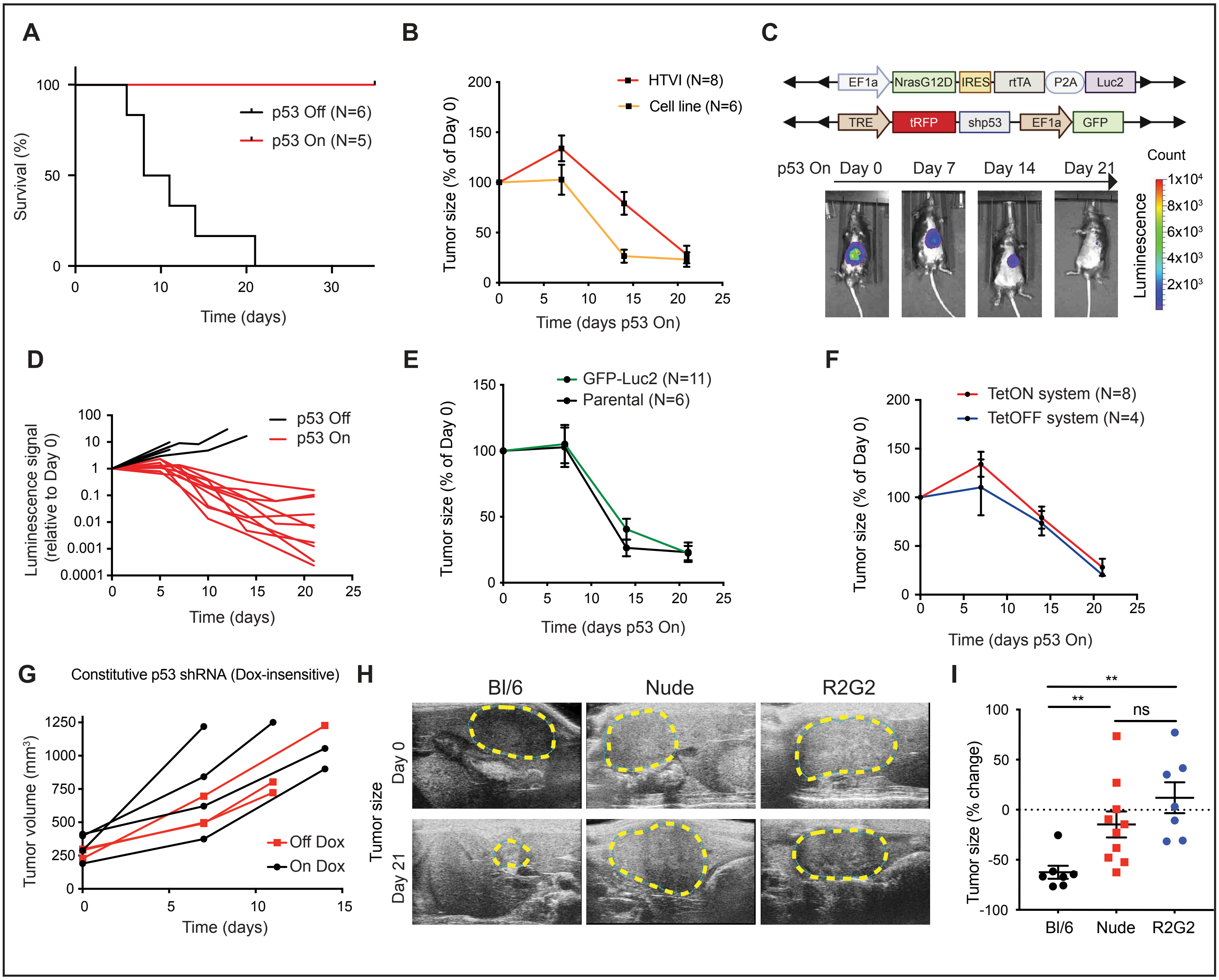

Supplementary Fig. S3. Characterization of senescence-induced tumor regression in NSP transplant model

A, Survival analysis of mice from orthotopic injection model of NSP tumor cells.
B, Comparison of tumor regression phenotype between HTVI and orthotopic injection model measured by ultrasound.
C, Representative bioluminescence images of tumor regression upon p53 restoration in the HTVI model using luciferase-containing transposon constructs. (Created with BioRender.com.)
D, Longitudinal tracking of tumor growth in orthotopic injection model. NSP cells were transduced with a GFP-luciferase vector to enable bioluminescence imaging. Each line represents an individual mouse. Related to Supplementary Fig S3A.
E, Comparison of tumor regression phenotype upon p53 restoration between parental and GFP-luciferase (GFP-Luc2) transduced tumor cells in orthotopic injection model.
F, Comparison of tumor regression phenotype upon p53 restoration between Tet-On (rtTA) and Tet-Off (tTA) system in the HTVI model.
G, Comparison the growth of tumor harboring a p53 hairpin driven by a constitutive promoter in the presence or absence of doxycycline (Dox) treatment. Each line represents an individual mouse.
H, Representative ultrasonogram of the tumor size at indicated time after restoring p53 expression in immunocompetent and -deficient mice.
I, Quantification of tumor size change between day 7 and 14 after p53 restoration from mice shown in (H) showing a trend to a greater defect of tumor regression in R2G2 compared to nudes.
Data is presented as mean ± s.e.m.

Supplementary Fig. S4. Senescence switches from immune-suppressive to immune-activated tumor microenvironment

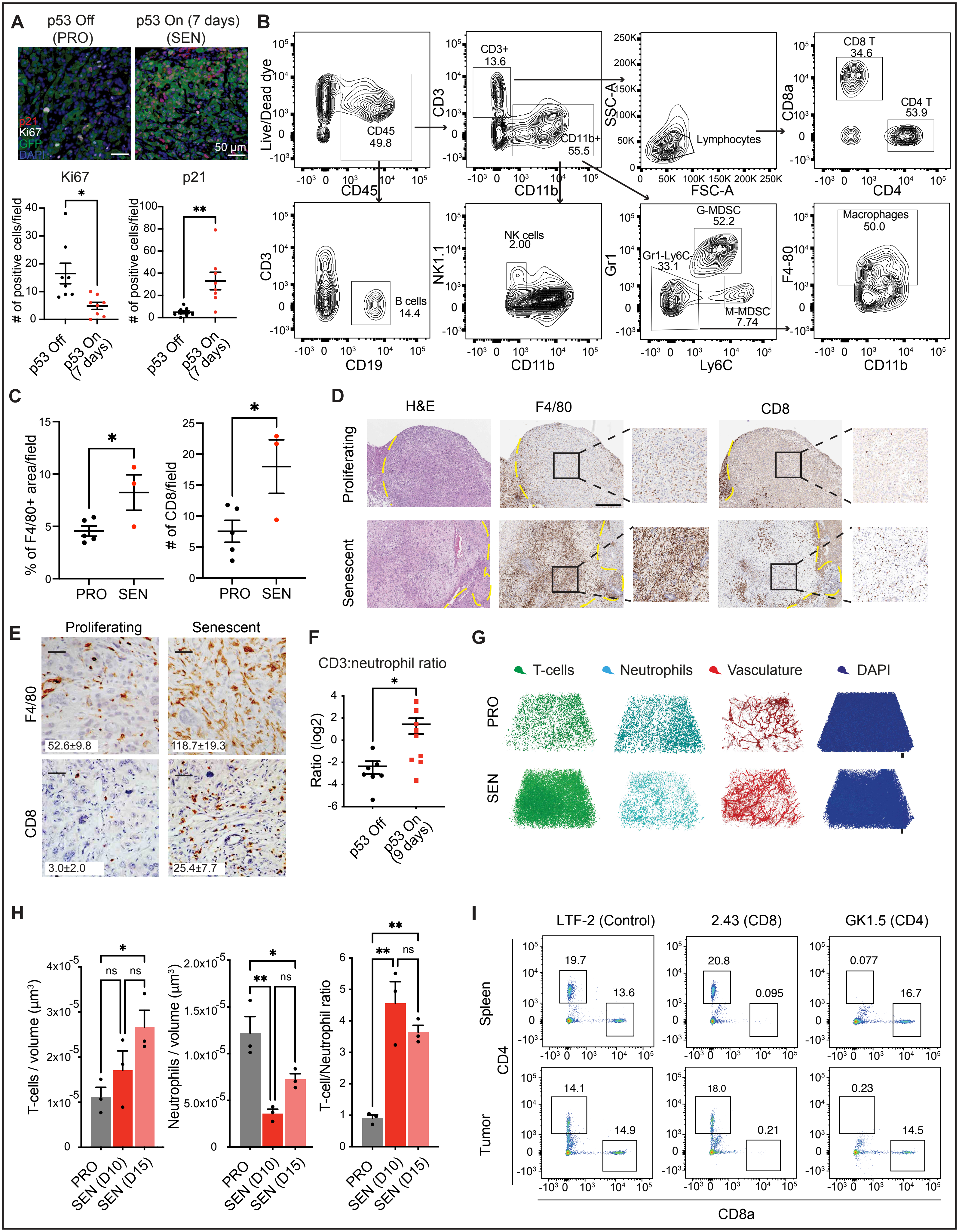

Supplementary Fig. S4. Senescence switches from immune-suppressive to immune-activated tumor microenvironment

A, Representative immunofluorescence images of Ki67 and p21 staining from p53 on (7 days) and off tumor sections. Quantification of images from 2 random fields per mouse and number was averaged. N=7 for p53 on (Dox withdrawal for 7 days) and N= 8 for p53 off group. Each dot represents a mouse. Related to Fig. 2A. Scale bar, 50 μm.

B, Gating strategy for immunophenotyping. Related to Fig. 2B.

C, Quantification of the percentage of F4/80+ area and the number of CD8 T cells per field in NSP tumor. 9-10 random fields are taken per mouse sample and number was averaged. Each dot represents a mouse. N=5 and 3 of the mice harboring p53-suppressed (PRO) or -restored tumor (SEN, Dox withdrawal for 9 days) respectively. Related to Fig. 2H.

D, Representative of H&E and IHC images of CD8 T cells and F4/80 positive macrophages staining in NSP transplantable tumor model. p53-on tumors (senescent) were collected at 9 days after Dox withdrawal. Note that some hepatocytes were stained with high background signal in CD8 staining, but they can be distinguished by morphological difference. Scale bar, 500 μm.

E, Representative IHC images of CD8 T cells and F4/80 positive macrophages staining in the HTVI-generated tumor. p53-restored tumors (senescent) were collected 14 days after randomization to indicated treatment. Quantification of number of cells per field from 2-3 random fields each mouse and N ≥ 4 mice per group. Scale bar, 100 μm.

F, CD3-to-neutrophil ratio calculated from flow cytometry measurements in Fig. 2B.

G, Individual channels of 3D imaging after tissue clearing from Fig. 2D. Scale bar, 100 μm.

H, Quantification of CD3 T cells and neutrophil density and the CD3-to-neutrophil ratio at the indicated time points of tumor collection (D, day). PRO, proliferating. SEN, senescent. Related to Fig. 2D.

I, representative flow cytometry plots of CD4 and CD8 T cells after depletion through corresponding antibodies.

Data is presented as mean ± s.e.m. A two-tailed student t-test is used. $^{**}p < 0.01$, $^{*}p < 0.05$.

Supplementary Fig S5. Single-cell characterization of senescence-induced cell shifts in CD8 T cells and myeloid cells

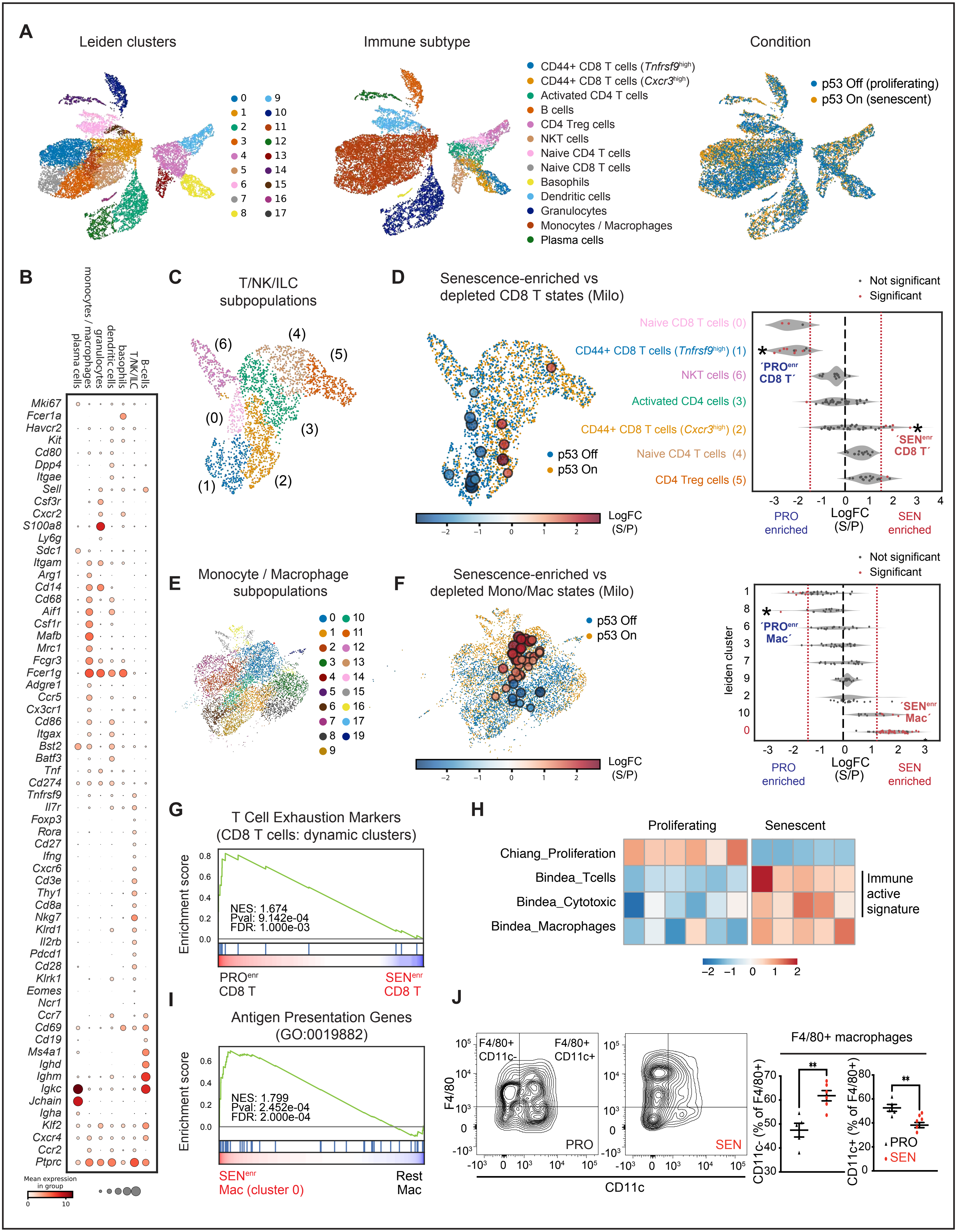

Supplementary Fig. S5. Single-cell characterization of senescence-induced cell shifts in CD8 T cells and myeloid cells

A, Clustering and immune subtyping of immune cells isolated from proliferating (p53 off) and senescent (p53 on) tumor lesions.

B, Expression of key immune marker genes by immune subtypes (see Fig. S5A)

C, Leiden clustering of T / NK / ILC cells.

D, Left panel, depletion (blue) and enrichment (red) of T / NK / ILC cell neighborhoods in senescent (p53 on) vs proliferating (p53 off) tumors. Right panel, log fold-change of T / NK / ILC cell neighborhoods in senescent (p53 on) vs proliferating (p53 off) tumors by clusters (see Fig. S5C). CD8 T cell clusters significantly enriched in proliferating (PROenr CD8 T) or senescent (SENenr CD8 T) tumors are marked with asterisks.

E, Leiden clustering of monocytes / macrophages.

F, Left panel, depletion (blue) and enrichment (red) of monocyte / macrophage neighborhoods in senescent (p53 on) vs proliferating (p53 off) tumors. Right panel, log fold-change of monocyte / macrophage neighborhoods in senescent (p53 on) vs proliferating (p53 off) tumors by clusters (see Fig. S5E).

G, GSEA comparing the expression T cell exhaustion markers (Pdcd1, Havcr2, Tox, Lag3, Ctla4, Tigit, Btla, Cd160, Ido1, Slamf6, Nt5e, Vsir, Cd276) between CD8 T cell clusters enriched in proliferating (p53 off; PROenr CD8 T) vs senescent (p53 on; SENenr CD8 T) tumors (as in Fig. S5D-right panel).

H, immune active signatures from publicly available dataset (2) and proliferation class signature (3).

I, GSEA comparing the expression of the indicated pathway between ´cluster 0´(F4/80high;CD11c-) macrophages (SENenr Mac) and the rest of cells in monocyte / macrophage compartment (shown in S5F-right panel).

J, Representative flow cytometry plot and quantification of macrophage population changes in proliferating and senescent tumor (Dox withdrawal for 9 days).

Supplementary Fig S6. Senescence-associated macrophages module CD8 T cell activation in vivo

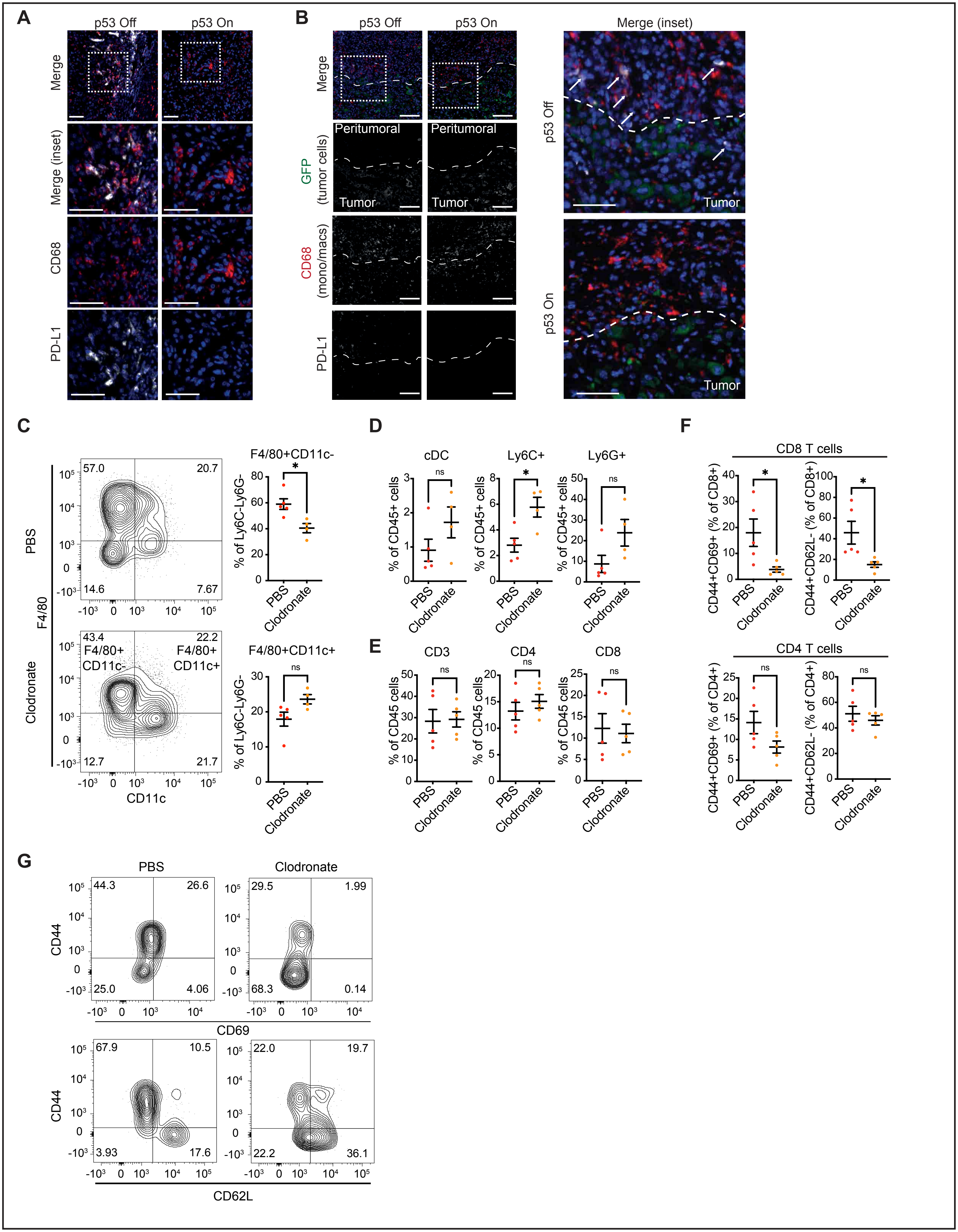

Supplementary Fig. S6. Senescence-associated macrophages module CD8 T cell activation in vivo

A and B, Representative immunofluorescence images of PD-L1 and CD68 (marker of macrophages) staining in tumor lesions (A). B, Overlay of PD-L1, CD68 and GFP (labeled tumor cells) showed negligible PD-L1 staining in senescent tumor (Dox withdrawal for 8 days).

C, Effect of liposomal clodronate treatment on macrophages population in tumors triggered to senesce (9 days after p53 restoration). Population changes of CD11b+Ly6G-Ly6C-F4/80+ macrophages and representative flow cytometry plots.

D, Effect of liposomal clodronate treatment on other myeloid lineages in tumors triggered to senesce. Population changes of conventional dendritic cells (cDC, CD11b-CD11c+MHC-II+CD103+), neutrophils/granulocytes (CD11b+Ly6G+) and monocytes (CD11b+Ly6C+).

E, Effect of liposomal clodronate treatment on lymphoid lineages in tumors triggered to senesce. Population changes of CD3, CD4 and CD8 T cells.

F and G, Activation state of CD8 and CD4 T cells upon liposomal clodronate treatment in tumors triggered to senesce (D). Representative flow cytometry plots (E).

Supplementary Fig. S7. Cell surfaceome is substantially remodeled in senescent cells

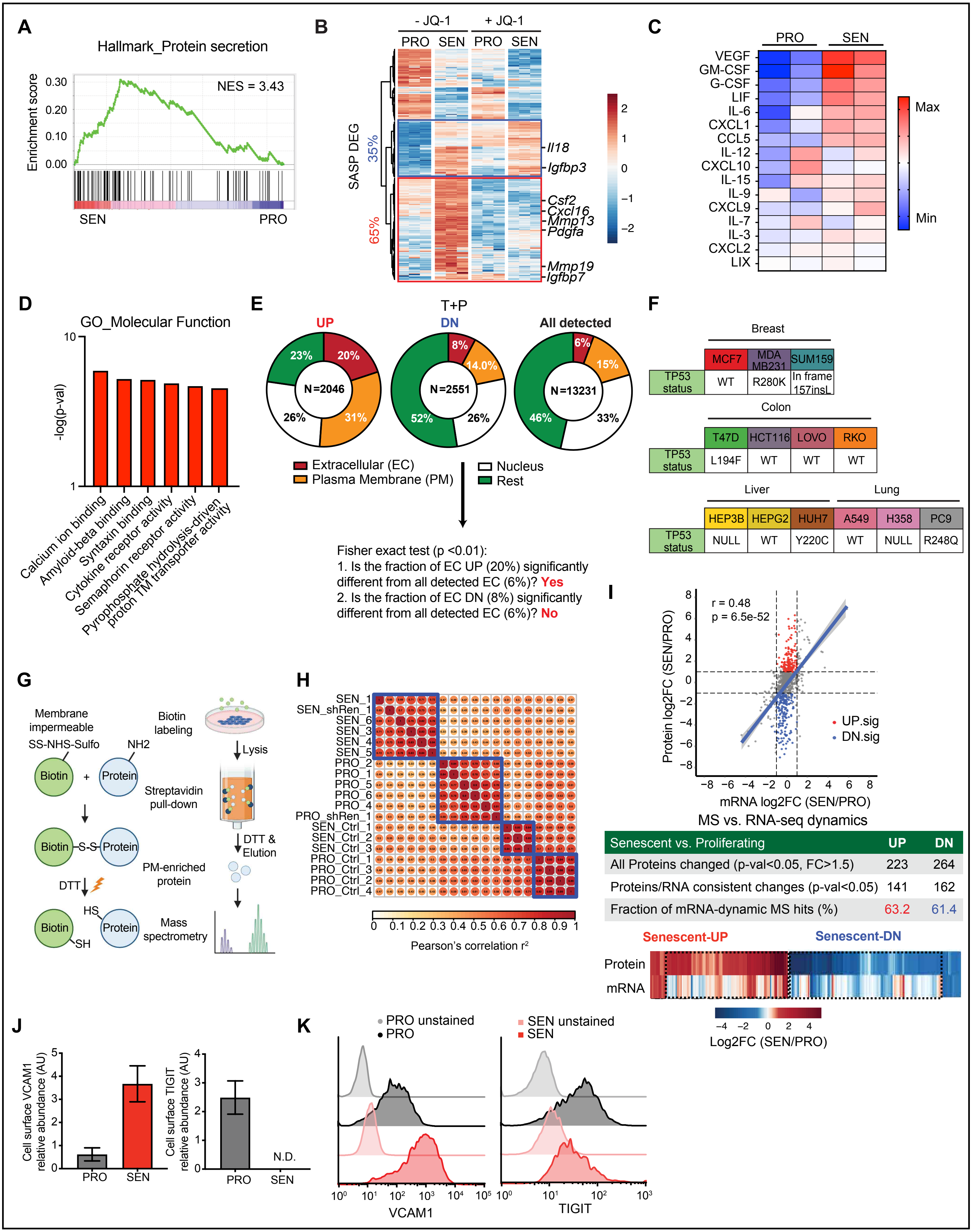

Supplementary Fig. S7. Cell surfaceome is substantially remodeled in senescent cells

A, GSEA (Hallmark) of RNA-Seq data from proliferating (PRO, p53 Off) vs. senescent (SEN, p53 On for 8 days) NSP liver tumor cells in vitro.

B, Transcriptomic analysis of differential expressed genes (DEGs) encoding secretory factors (SASP factors) in NPS cells in the presence or absence of JQ-1 treatment.

C, Cytokine array of conditioned medium collected from proliferating and senescent (p53 On Day 6 to Day 8) cells in vitro. Samples are from 2 independent biological replicates.

D, GO analysis of DEGs encoding plasma membrane proteins upregulated in senescent cells. Related to Fig. 3C

E, Top panel, Subcellular localization of detected DEGs (TPM > 1; $p < 0.05$; fold change > 2) from in vitro NSP cells treated with trametinib/palbociclib (T+P) or vehicle. Bottom panel, Calculation of p value of Fisher exact test using data from the supplementary Fig. S4E. A more stringent criteria for statistical significance is used, $p < 0.01$. Related to Fig. 3E.

F, p53 mutation status of human cell lines used in SENESCopedia. Related to Fig. 3E.

G, Graphic illustration of the protocol of plasma membrane-enriched mass spectrometry (MS).

H, Correlation plot of MS samples. (Created with BioRender.com.)

I, Left panel, XY plot of total proteins profiled by MS against corresponding transcriptomic expression profiled by RNA-seq. Right panel, summary of MS profiling and RNA-seq comparison

J and K, validation of two MS hits using flow cytometry. PRO, proliferating. SEN, senescent. N.D., not detected. Data is presented as mean ± s.e.m.

Supplementary Fig. S8. Cell-intrinsic sensitization to IFN-γ in senescent cells independent of p53 status

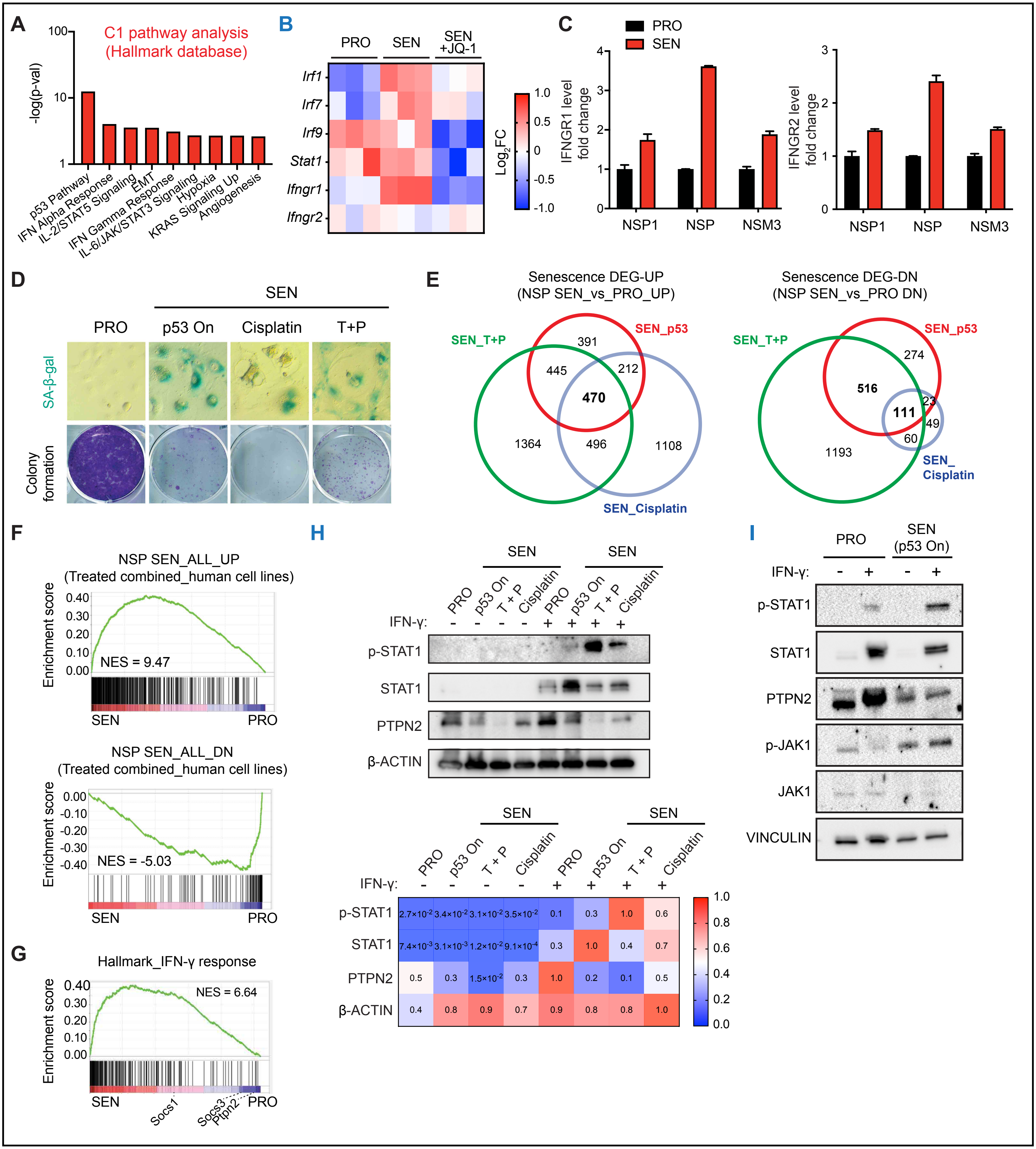

Supplementary Fig. S8. Cell-intrinsic sensitization to IFN-γ in senescent cells independent of p53 status

A, Pathway analysis of cluster 1 (C1, senescence-specific) shown in Fig. 3D against MsigDB Hallmark genesets.

B, mRNA expression of positive IFN-γ signaling mediators in proliferating and senescent NSP cells and senescent cells treated with JQ-1. Relative mRNA expression is normalized to geometric means of mRNA reads from all samples.

C, IFNGR1 and IFNGR2 level validated in 3 independent p53-restorable liver cancer cell lines. NSP is predominantly used in this study.

D, SA-β-gal staining of NSP cells treated with different senescence triggers.

E, Overlapping DEGs from RNA-seq in NSP cells treated with different senescence triggers to identify common signatures upregulated (UP) and downregulated (DN) in proliferating (PRO) vs. senescent (SEN) cells, which is composed of 111 and 470 genes respectively. p53, p53 restoration; T+P, trametinib plus palbociclib.

F, GSEA of the combined RNA-seq results from all human cell lines trigger to senesce in SENESCopedia showing enrichment of genes upregulated (ALL_UP) or downregulated (ALL_DN) in our common senescence signature from (D).

G, GSEA of same RNA-seq results in (E) showing an enrichment in MSigDB Hallmark IFN-γ response pathway in senescent cells.

H and I, Independent immunoblot analysis of NSP cells under different senescence triggers, in the presence or absence of IFN-γ (1 ng/ml) (H). Immunoblot analysis of JAK-STAT signaling activation upon senescence and IFN-γ treatment (I). Related to Fig. 4E.

Data is presented as mean ± s.e.m. Two-tailed student t-test is used.

Supplementary Fig. S9. IFN-γ does not induce senescence in proliferating NSP tumor cells.

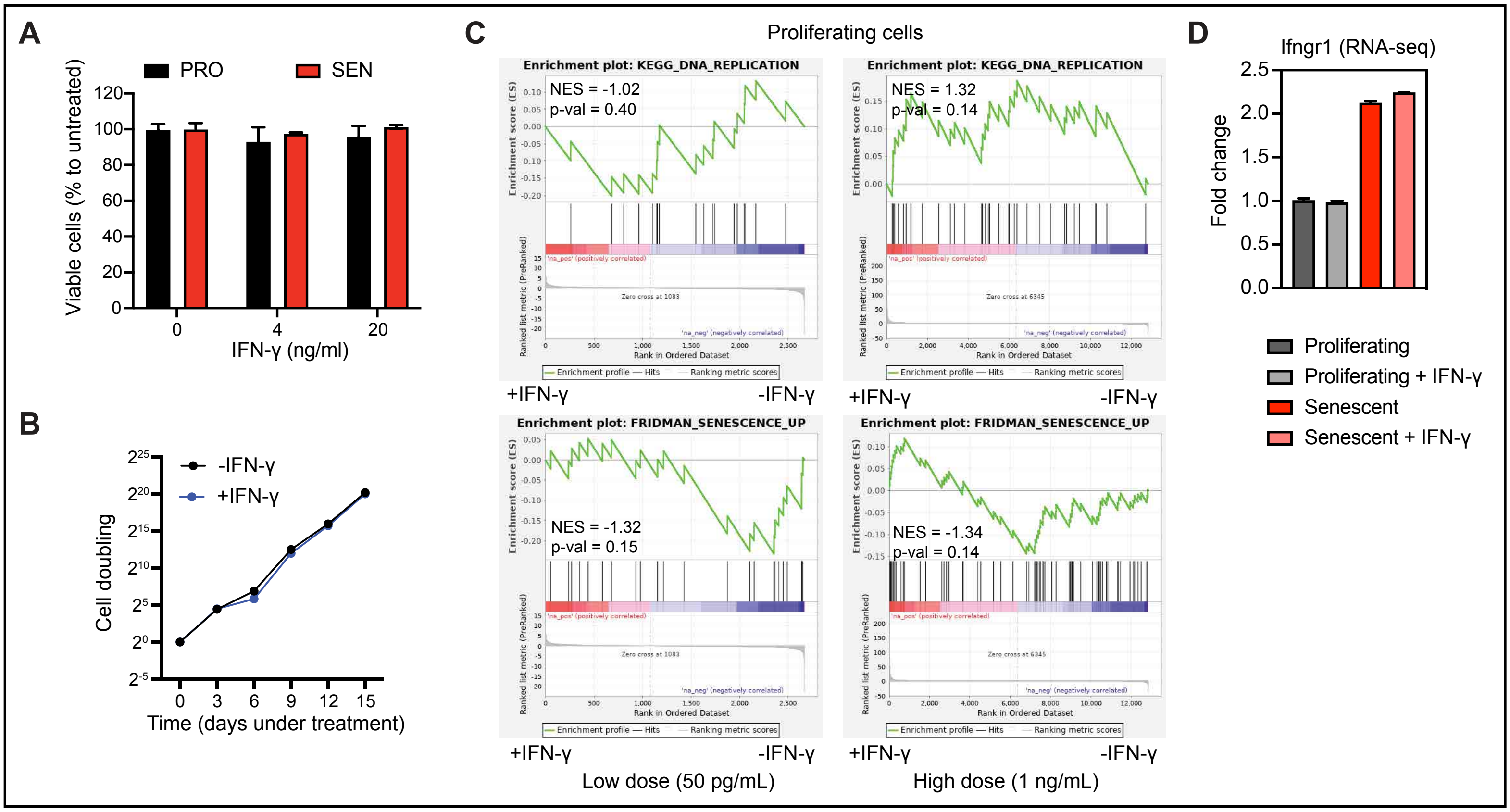

Supplementary Fig. S9. IFN-γ does not induce senescence in proliferating NSP tumor cells

A, Viability assay of proliferating and p53-restored senescent NSP cells treated with indicated dose of IFN-γ for 48 hours.

B, Comparison of cell doublings in proliferating NSP tumor cells treated with or without IFN-γ (1ng/ml). Experiment is performed in triplicate wells.

C, GSEA of proliferating NSP cells treated with or without IFN-γ at low (50 pg/ml) or high (1 ng/ml) dose for 24 hours against selected MSigDB signatures.

D, mRNA expression of Ifngr1 in proliferating and senescent NSP cells upon IFN-γ treatment (50 pg/ml).

Supplementary Fig. S10. Overexpression of IFNGR1 alone is insufficient to sensitize proliferating NSP cells to IFN-γ

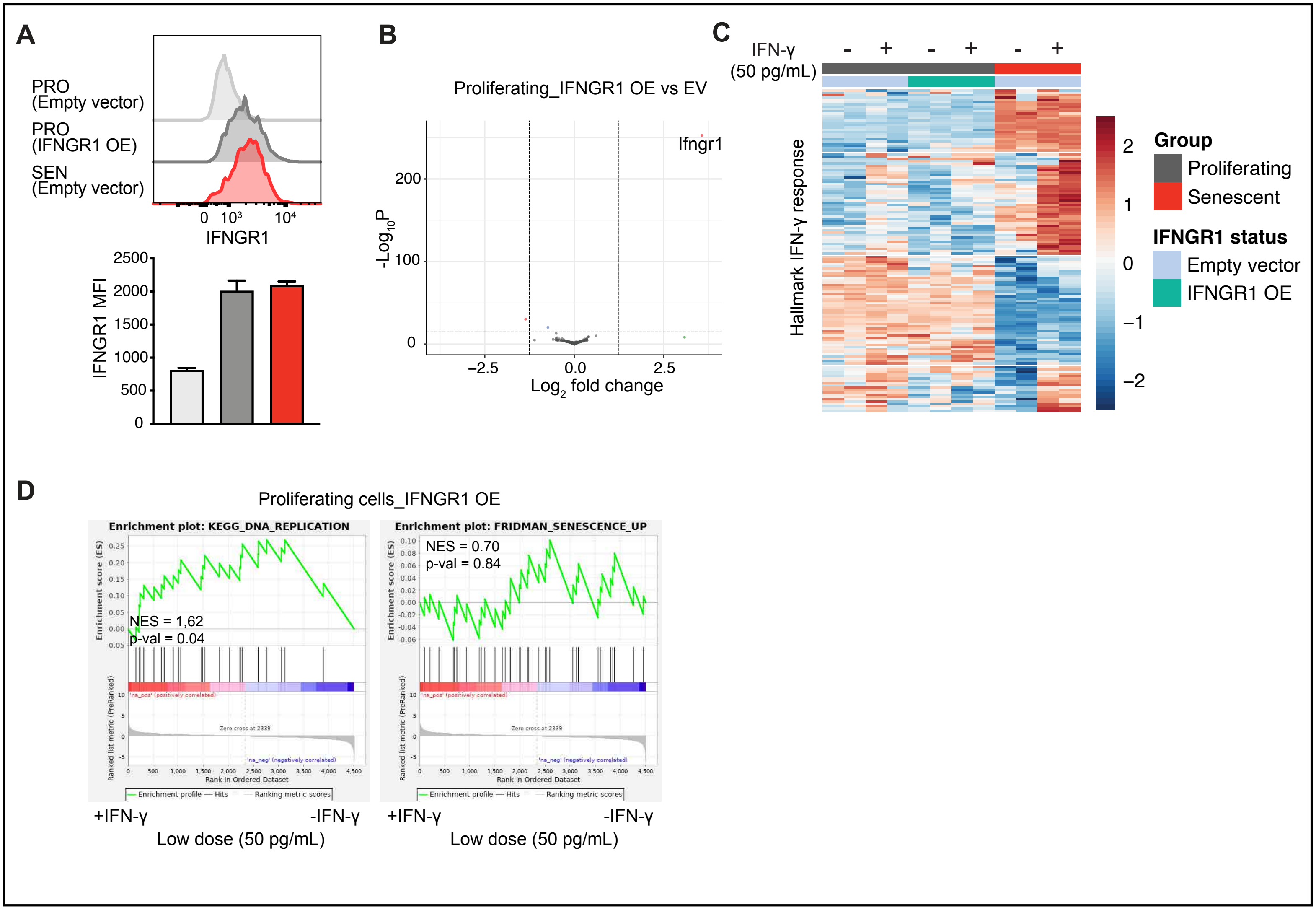

Supplementary Fig. S10. Overexpression of IFNGR1 alone is insufficient to sensitize proliferating NSP cells to IFN-γ

A, Level of cell surface IFNGR1 in proliferating cells +/- IFNGR1 overexpression (OE) and senescent cells.

B, Volcano plot of transcriptomic analysis comparing proliferating cells overexpressing IFNGR1 and empty vehicle (EV).

C, Transcriptomic analysis of proliferating cells +/- IFNGR1 overexpression and senescent cells treated with IFN-γ (50 pg/ml) for 24 hours.

D, GSEA of RNA-seq from IFNGR1-overexpressing proliferating cells treated with or without IFN-γ for 24 hours.

Supplementary Fig. S11. Cooperativity between senescence and IFN-γ signaling in inducing surface HLA in human cell lines

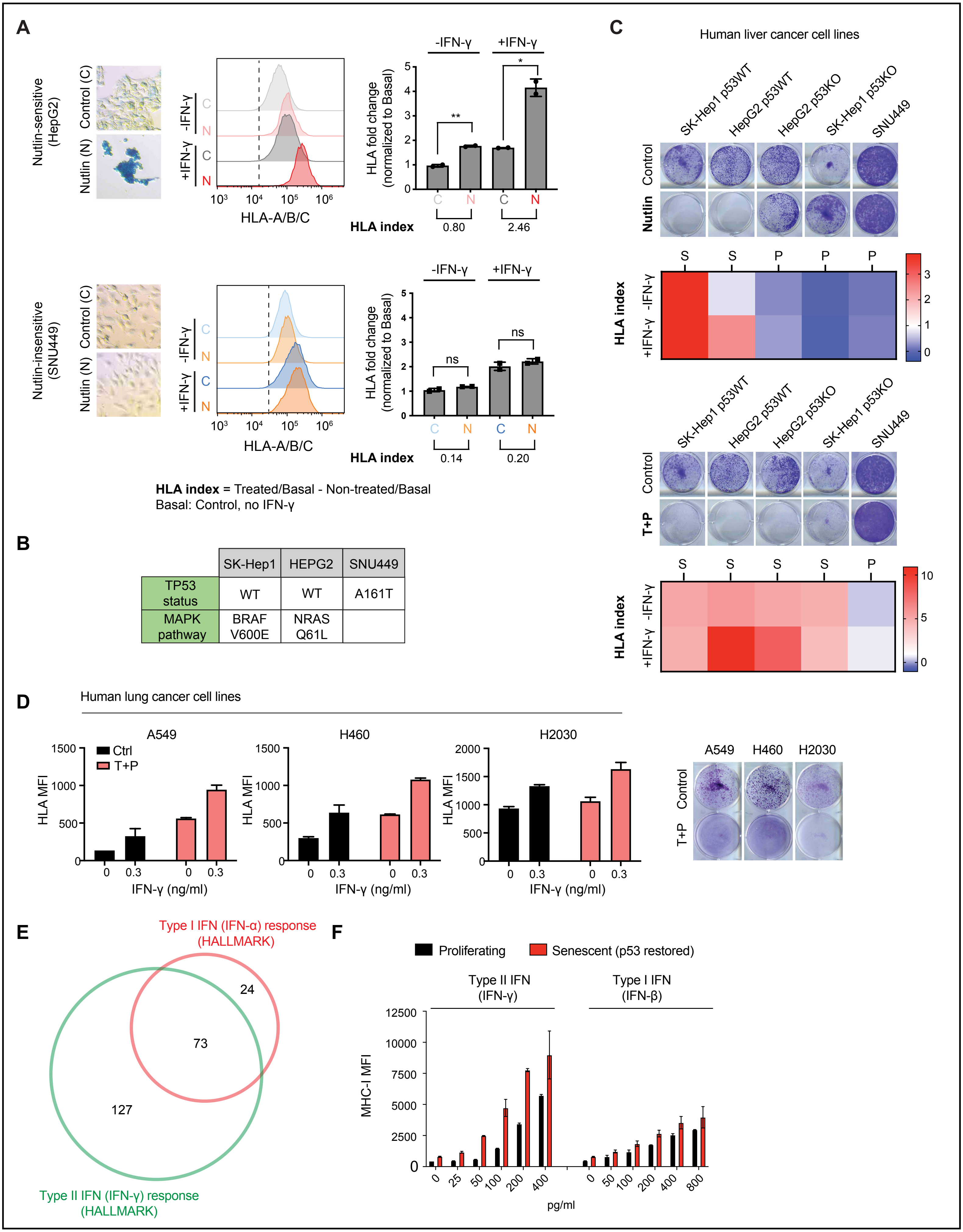

Supplementary Fig. S11. Cooperativity between senescence and IFN-γ signaling in inducing surface HLA in human cell lines

A, Human liver cancer cell lines and isogenic p53 KO clones were treated with indicated drugs to induce senescence. Cells were treated with human recombinant IFN-γ (1 ng/ml) and HLA-A/B/C was measured after 24h of treatment. "HLA-index" was determined by calculating the HLA level difference between drug-treated vs. untreated cells, in the presence or absence of IFN-γ treatment. Here shown is an example of HLA-index calculation.

B, p53 and RAS pathway mutation status of the human cell lines used in this experiment.

C, The summary of 5 human cell lines (including two isogenic p53 KO clones) treated with nutlin or trametinib + palbociclib (T+P).

D, Left panel, cell surface HLA in human lung cancer lines treated with vehicle control or T+P +/- IFN-γ treatment for 24 hours. Right panel, colony formation assay of indicated cell lines previously treated with vehicle control or T+P and plated for 7 days.

E, Venn diagram of the gene list from Hallmark IFN-γ (Type II IFN) and IFN-α (Type I IFN) response.

F, Comparison of MHC-I level in proliferating and senescent NSP cells treated with indicated doses of IFN-γ or IFN-β for 24 hours.

S, senescent; P, proliferating. Data is presented as mean ± s.e.m.

Supplementary Fig. S12. Cell-state and microenvironmental context shape IFN-γ pathway activation

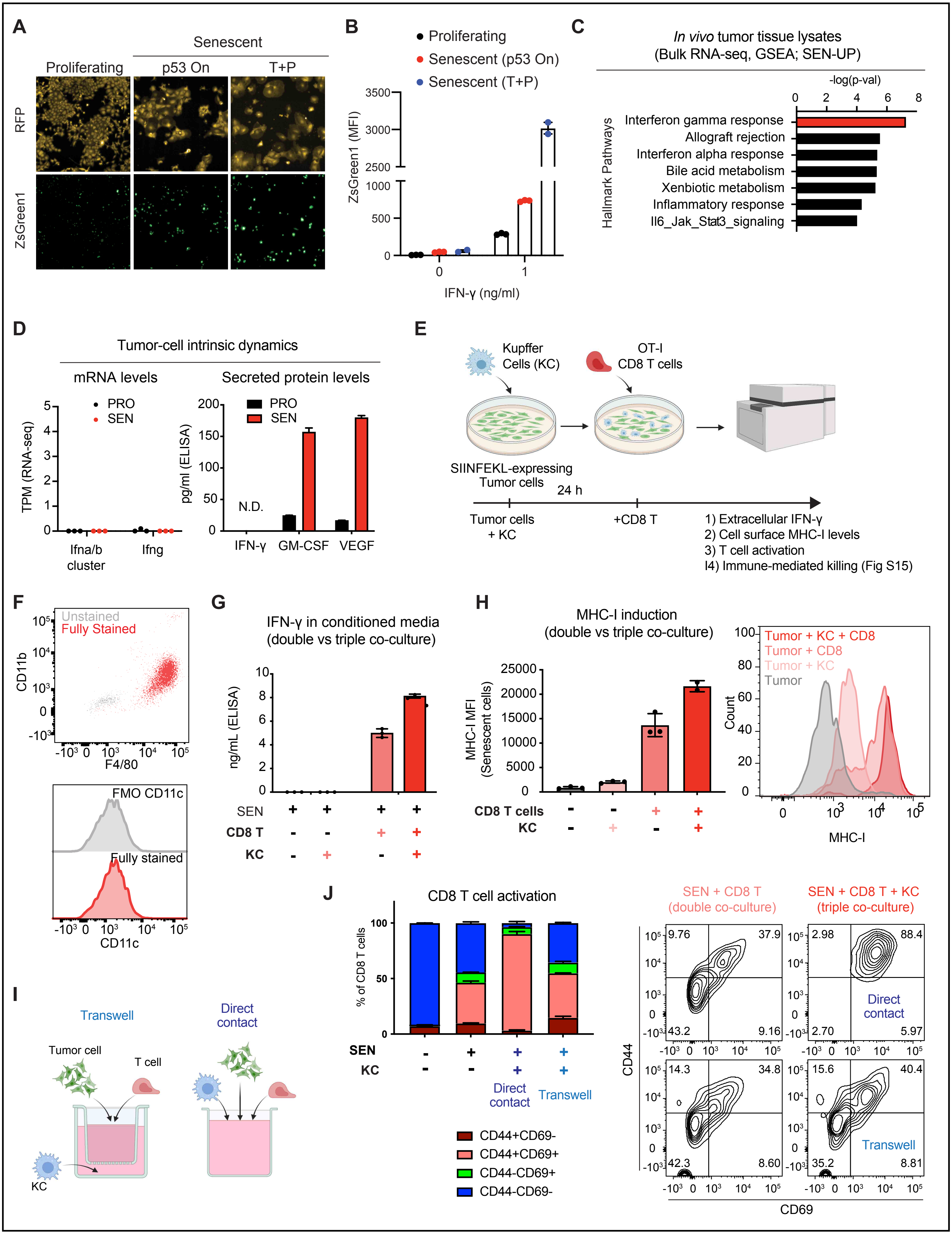

Supplementary Fig. S12. Cell-state and microenvironmental context shape IFN-γ pathway activation

A, Representative microscopic images of IGS reporter-expressing proliferating and senescent NSP tumor cells triggered by p53 restoration or trametinib + palbociclib (T+P) in combination with IFN-γ (1 ng/ml) treatment.

B, Quantification of ZsGreen1 intensity from (A).

C, GSEA (Hallmark) of RNA-Seq data from in vivo bulk tumor generated by HTVI comparing proliferating and senescent (p53 On, Dox withdrawal for 12 days) samples.

D, Left panel, transcripts of indicated genes from RNA-seq of proliferating and senescent NSP cells in vitro. Right panel, cytokine array of conditioned medium collected from proliferating and senescent (p53 On Day 6 to Day 8) NSP cells in vitro. Related to Fig. 6E.

E, Schematic of co-culture experimental setup. OT-I T cells (T), Kupffer cells (KC) and SIINFEKL-expressing NSP cells. GFP-expressing tumor cells were plated followed by addition of KC 6-8 hours later. Deep red dye-labeled OT-I T cells were added 24 hours after tumor cells were plated. Related to Fig. 6I. (Created with BioRender.com.)

F, Purity of isolated KC cells examined by flow cytometry. Related to (E).

G, Cytometric bead array (CBA) assay for probing IFN-γ level from conditioned medium of in vitro co-culture assay collected at 48 hours after triple cell types co-culture starts. Related to (E).

H, MHC-I level of senescent cells in the triple cell types co-culture experiment. Cells were harvested at 48 hours after triple cell types co-culture starts. Related to (E).

I, Schematic of triple cell types co-culture in transwell or same well.

J, Activation status of CD8 T cells with or without direct contact with macrophages in triple cell types co-culture experiment. Related to (I).

MFI, median fluorescence intensity. Data is presented as mean ± s.e.m.

Supplementary Fig. S13. Induction of senescence in tumor cells is independent of IFN-γ signaling

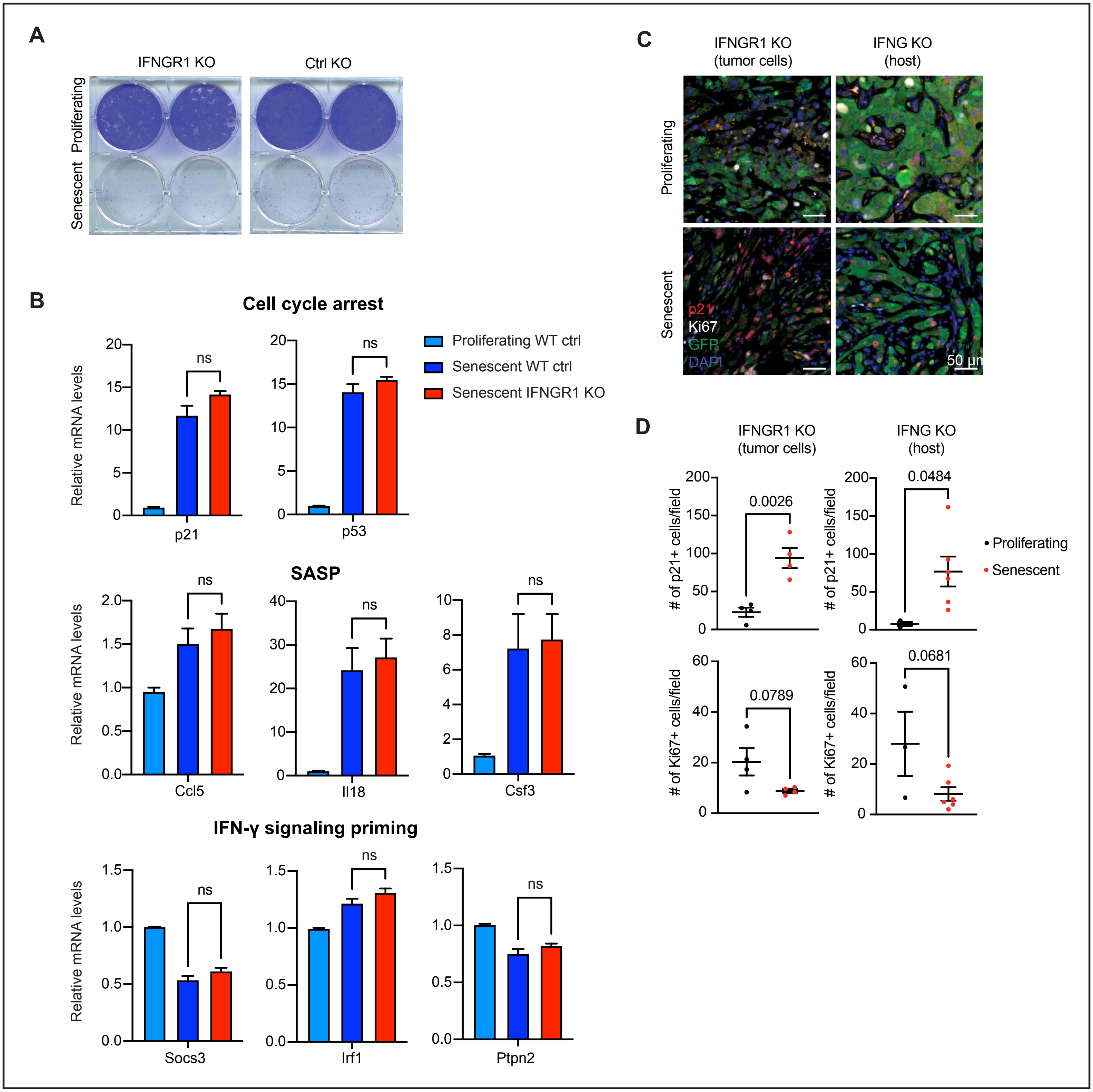

Supplementary Fig. S13. Induction of senescence in tumor cells is independent of IFN-γ signaling
A, Colony formation assay of IFNGR1 KO and WT NSP cells.
B, RT-qPCR of selected senescence-related genes and IFN-γ signaling genes in WT proliferating, senescent and IFNGR1 KO senescent cells.
C and D, Representative immunofluorescence images of p21 and Ki67 staining in tumor sections of the corresponding perturbation condition (C). D, Quantification of the number of p21 and Ki67 positive cells per field. 4 random fields were taken per mouse and the numbers were averaged. Each dot represents a mouse.

Supplementary Fig. S14. Blunting IFNGR1/IFN-γ signaling in tumor cells does not affect senescence phenotype

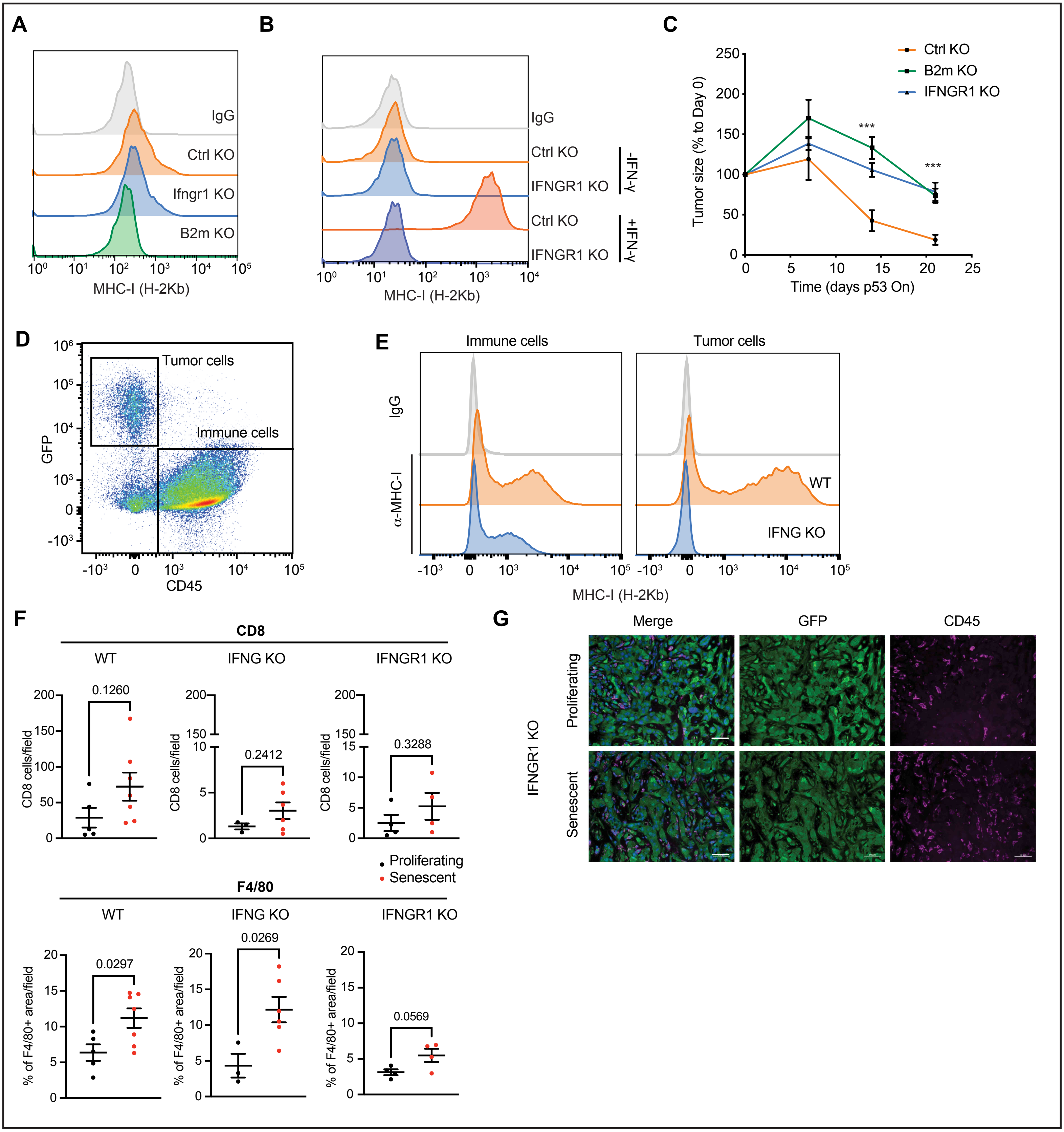

Supplementary Fig. S14. Blunting IFNGR1/IFN-γ signaling in tumor cells does not affect senescence phenotype

A, Flow cytometry analysis of MHC-I level in Ctrl, Ifngr1 and B2m KO tumor cells at the basal level.

B, Flow cytometry analysis of MHC-I level in Ifngr1 KO and control sgRNA (Ctrl KO) tumor cells treated with IFN-γ (1ng/ml).

C, Tumor regression phenotype of Ctrl, Ifngr1 and B2m KO tumor upon p53 restoration. Figure was overlay with Fig. 7B.

D, Representative flow cytometry plots gating the GFP+ tumor cells and CD45+ immune cells. Related to Fig. 7E.

E, Representative flow cytometry plots showing MHC-I level in tumor cells and immune cells from WT and IFNG KO mice.

F, Quantification of the percentage of F4/80+ area and the number of CD8 T cells per field. 4 random fields were taken per mouse and the numbers were averaged. Each dot represents a mouse.

G, Representative immunofluorescence of Ifngr1 KO tumor in Bl/6N mice. Related to Fig. 7F. Scale bar, 50 μm

Data is presented as mean ± s.e.m. Two-tailed student t-test is used. $*p < 0.05$; $**p < 0.01$; $***p < 0.001$.

Supplementary Fig. S15. CD8 T cells and macrophages cooperate to kill senescent tumor cells in a IFN-γ -dependent manner

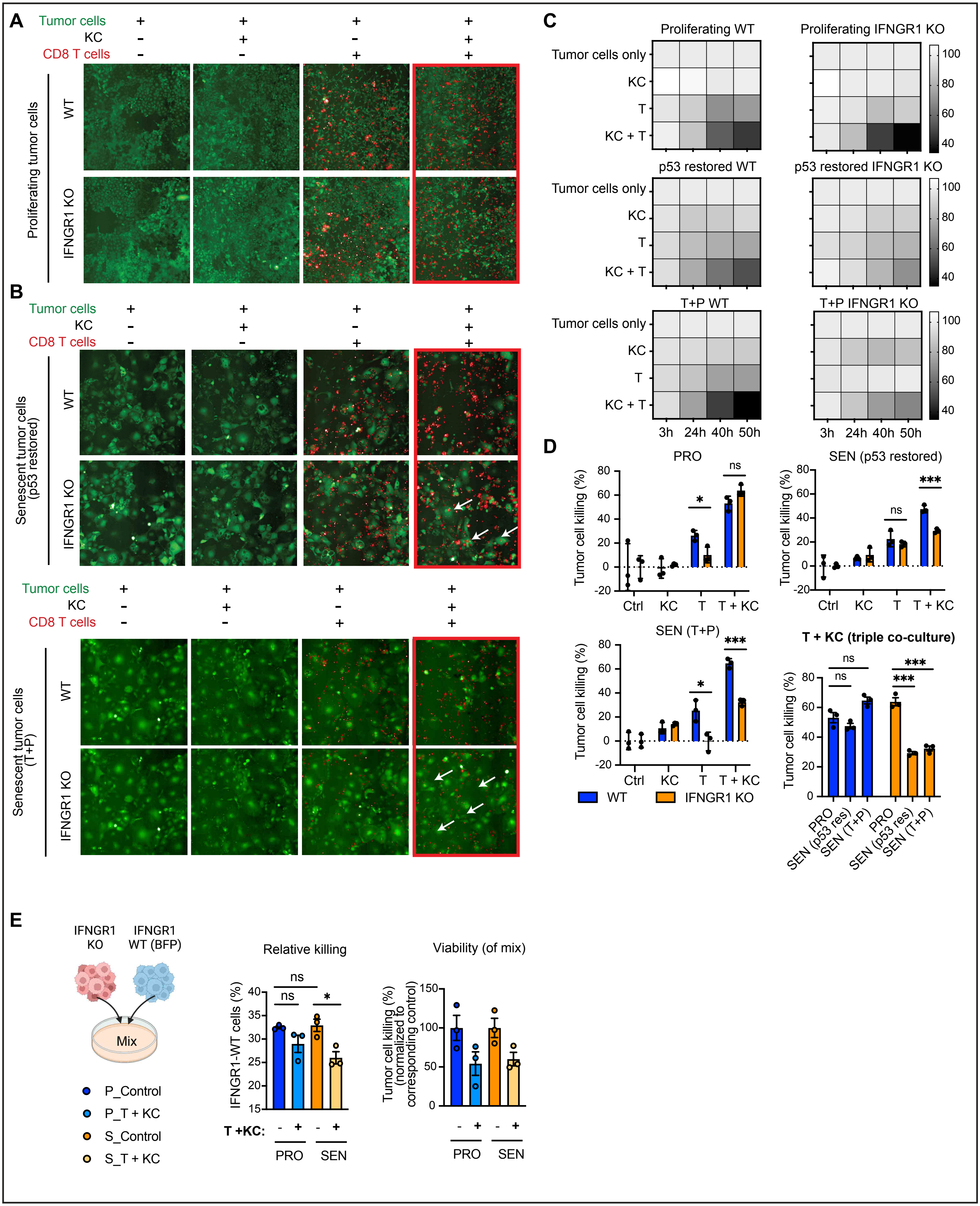

Supplementary Fig. S15. CD8 T cells and macrophages cooperate to kill senescent tumor cells in an IFN-γ -dependent manner

In vitro co-culture assay of SIINFEKL-expressing NSP tumor cells, macrophages (kupffer cells, KC) and OT-I CD8 T cells. See also supplementary figure. S12E for experimental setup. The ratio of KC:T:tumor cells is 40:10:1.

A and B, Representative images correspond to the end time point (50 hours) of co-culture assay, and arrows highlight the persistence of senescent (but not proliferating) tumor cells perturbed for IFN-γ signaling. Images of proliferating cells (A) and senescent cells (p53 restored and trametinib+palbociclib [T+P] treated (B) labeled in green and T cells labeled in red.

C and D, Quantification of viable proliferating and senescent cells at the indicated time points. Percentage of tumor cell killing is calculated by measuring the changes of GFP positive cell number normalized to the untreated (no immune cells) control at the indicated time points, as assessed with the InCell high-content microscope using n= 3 independent wells per experimental condition. Bar graph of tumor cell killing at the end time point (50 hour) co-culture assay (D).

E, Competition assay of IFNGR1-expressing (WT) and KO cells in the triple co-culture setting. Left, schematic of mixing assay: IFNGR1 WT and KO cells were first mixed at 1:2 ratio and kept on Dox (P) or off Dox for 6 days (to induce senescence, S) followed by addition of KC and CD8 T (T) cells. Mixtures non-exposed to immune effector cell types were included as controls. Right, Percentages of IFNGR1 WT (of total cells) in Proliferating (PRO) or Senescent (SEN) mixtures measured by flow cytometry. Note IFNGR1 WT senescent cells are selectively eliminated overtime –an effect that requires the presence of immune effector cell types (i.e. not driven by senescence induction per se, middle panel) and that is less pronounced in proliferating counterparts, even upon normalization of total killing (right panel). (Created with BioRender.com.)

Supplementary Fig. S16. Graphic illustration of our working model

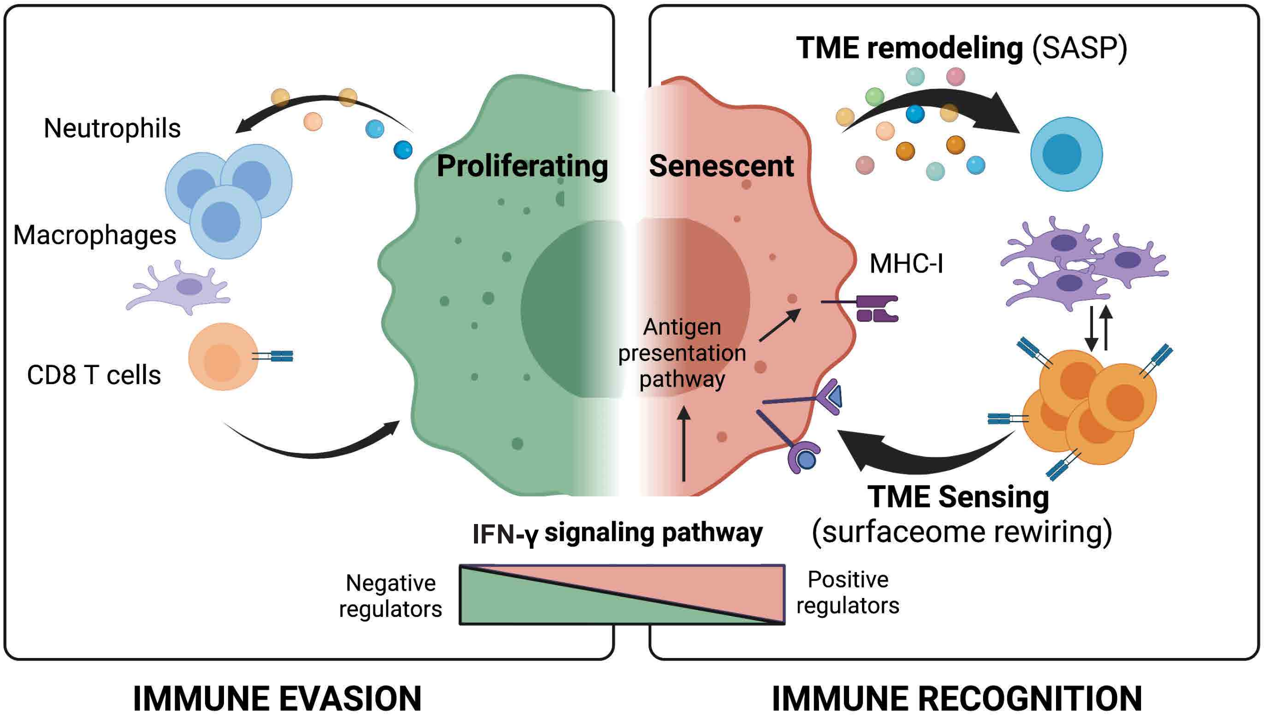

Supplementary Fig. S16. Graphic illustration of our working model
(Created with BioRender.com.)

SUPPLEMENTARY TABLES

Supplementary Table S1: differential expression analyses of CD8 T and macrophages populations of proliferating (p53 off) vs. senescent (p53 on) tumors by scRNA-seq.

Supplementary Table S2: RNA-Seq data of proliferating (PRO) or senescent (SEN) NSP liver tumor cells, for both p53-restoration and drug-induced (trametinib+palbociclib) settings. PRO and SEN cells were also treated with the BET inhibitor JQ-1 (500 n, 48 h), to expose BRD4-mediated transcriptional output in each cellular state.

Supplementary Table S3: low (50 pg/ml) and high (1 ng/ml) dose of IFN-γ treatment in proliferating and senescent NSP cells.

References

1. Llovet JM, Kelley RK, Villanueva A, Singal AG, Pikarsky E, Roayaie S, et al. Hepatocellular carcinoma. Nat Rev Dis Primers 2021;7(1):6 doi 10.1038/s41572-020-00240-3.

2. Bindea G, Mlecnik B, Tosolini M, Kirilovsky A, Waldner M, Obenauf AC, et al. Spatiotemporal dynamics of intratumoral immune cells reveal the immune landscape in human cancer. Immunity 2013;39(4):782-95 doi 10.1016/j.immuni.2013.10.003.

3. Chiang DY, Villanueva A, Hoshida Y, Peix J, Newell P, Minguez B, et al. Focal gains of VEGFA and molecular classification of hepatocellular carcinoma. Cancer Res 2008;68(16):6779-88 doi 10.1158/0008-5472.CAN-08-0742.
